# Caffeine inhibits hypothalamic A_1_R to excite oxytocin neuron and ameliorate dietary obesity in mice

**DOI:** 10.1038/ncomms15904

**Published:** 2017-06-27

**Authors:** Liufeng Wu, Jia Meng, Qing Shen, Yi Zhang, Susu Pan, Zhuo Chen, Ling-Qiang Zhu, Youming Lu, Yuan Huang, Guo Zhang

**Affiliations:** 1 Key Laboratory of Environmental Health, Ministry of Education, Department of Toxicology, School of Public Health, Tongji Medical College, Huazhong University of Science and Technology, Wuhan, Hubei 430030, China; 2 Institute for Brain Research, Collaborative Innovation Center for Brain Science, Huazhong University of Science and Technology, Wuhan, Hubei 430030, China; 3 Department of Pathophysiology, School of Basic Medicine, Tongji Medical College, Huazhong University of Science and Technology, Wuhan, Hubei 430030, China; 4 Department of Physiology, School of Basic Medicine, Tongji Medical College, Huazhong University of Science and Technology, Wuhan, Hubei 430030, China

## Abstract

Caffeine, an antagonist of the adenosine receptor A_1_R, is used as a dietary supplement to reduce body weight, although the underlying mechanism is unclear. Here, we report that adenosine level in the cerebrospinal fluid, and hypothalamic expression of A_1_R, are increased in the diet-induced obesity (DIO) mouse. We find that mice with overexpression of A_1_R in the neurons of paraventricular nucleus (PVN) of the hypothalamus are hyperphagic, have glucose intolerance and high body weight. Central or peripheral administration of caffeine reduces the body weight of DIO mice by the suppression of appetite and increasing of energy expenditure. We also show that caffeine excites oxytocin expressing neurons, and blockade of the action of oxytocin significantly attenuates the effect of caffeine on energy balance. These data suggest that caffeine inhibits A_1_Rs expressed on PVN oxytocin neurons to negatively regulate energy balance in DIO mice.

## Abstract (web summary)

The mechanism by which caffeine, an antagonist of adenosine receptors, regulates metabolism is not clear. Here the authors show that adenosine A_1_R receptor expression is increased in the hypothalamus of diet-induced obesity mice, and that body weight can be alleviated by central administration of caffeine via its action on hypothalamic oxytocin neurons.

In the United States, the overall prevalence of obesity in adults reached 37.7% during the years 2013–2014 (ref. 1). The population of obese individuals has also been increasing rapidly in the developing countries. For example, in China, the percentage of obese adults has increased from 3.6% in 1992 to 12.2% in 2012 (ref. 2). Obesity is a risk factor for type 2 diabetes, cardiovascular disease and certain types of cancers. Currently, medical treatment of obesity includes pharmacological and surgical approaches^3^. Only three drugs have been approved by FDA for long-term management of obesity^3^.

Consumption of caffeine (1,3,7-trimethylxanthine), one of the active ingredients in coffee, tea and soft drinks, has been linked to the long-term reduction of body weight gain^4^, however, the underlying mechanisms remain largely unknown. Caffeine is a recognized antagonist for adenosine receptor, which includes 4 subtypes in mammals: A_1_R, A_2A_R, A_2B_R and A_3_R. Adenosine receptors are G protein-coupled receptors, with A_1_R and A_3_R mainly coupled to the inhibitory G_i_ or G_o_ protein, and A_2A_R as well as A_2B_R mostly coupled to the stimulatory G_s_, G_q_ or G_olf_ protein^5^,^6^,^7^,^8^. In addition, adenosine receptors could regulate other intracellular signalling molecules, such as mitogen-activated protein kinase, to modulate cell physiology^6^. A_1_R is widely distributed in the body, with particularly high level of expression in the brain. A_2A_R is abundantly expressed in the striatum^5^,^7^. A_2B_R is also widely expressed throughout the body, but the overall abundance is low. A_3_R is expressed at low levels in most tissues.

Adenosine, the natural agonist for adenosine receptors, is a prototypic neuromodulator^9^. In the nervous system, adenosine acts on A_1_R to suppress neuronal activity, mainly through the regulation of downstream signalling molecules, such as the inhibition of protein kinases A and C, phospholipase C, calcium channels and activation of potassium channels^5^,^6^,^7^,^8^. In contrast, activation of A_2A_R or A_2B_R stimulates neuronal activity by increasing the activity of protein kinase A and/or mitogen-activated protein kinase^5^,^6^,^7^,^8^. Adenosine receptor is involved in an array of physiological and pathological processes, including memory, sleep, anxiety, aggression, locomotion, pain, cardiac and immune functions, as well as neurodegenerative diseases^5^,^10^. However, the precise role of neuronal adenosine receptor in energy balance remains less well understood.

The hypothalamus is the central regulator of energy balance in animals. Previously, studies have identified several key hypothalamic nuclei that are involved in the regulation of energy homeostasis (for example, paraventricular nucleus (PVN), arcuate (Arc), ventromedial and dorsomedial (DMH) nuclei)^11^,^12^. Several neuropeptides synthesized and released from the neurons in these nuclei, such as oxytocin (Oxt) in the PVN, Agouti-related peptide (AgRP) and α-melanocyte-stimulating hormone in the Arc, have also been identified as the key neurotransmitters regulating energy balance^11^,^12^. In these neuropeptide-synthesizing neurons, studies have discovered several key genes and signalling pathway that are involved in energy balance and/or the pathogenesis of dietary obesity, such as, PTP1b (ref. 13), IKKβ (ref. 14), ER stress^14^,^15^, JNK1 (ref. 16), GABA (ref. 17), Synaptotagmin-4 (ref. 18), PPARγ (refs 19, 20), NOS1 (ref. 21), Mitofusin^22^ and P2Y6 (ref. 23). However, it is still unclear whether neuronal adenosine receptor is involved in the regulation of these neuropeptides.

Here, we show that there are aberrations of the adenosine receptor signalling in the hypothalami of diet-induced obesity (DIO) mice. Mice with ectopic expression of A_1_R in the PVN neurons gained more body weights and consumed more foods than controls. Central or peripheral caffeine treatment reduces the body weights of DIO mice. Moreover, caffeine treatment improves glucose intolerance and lipid outcomes in these mice. Central or peripheral administration of caffeine excites neurons in the PVN. Lastly, we show that Oxt mediates, whereas blockade of the action of Oxt, but not arginine vasopressin (AVP) or thyrotropin releasing hormone (TRH), significantly attenuates caffeine’s effect of negatively regulating energy balance. We have therefore uncovered a role for hypothalamic adenosine signalling in the regulation of energy balance, and found that caffeine regulates energy metabolism by relieving A_1_R-mediated inhibition of PVN Oxt neurons.

## Results

### Elevated levels of adenosine in the DIO mice

**Figure 1:**
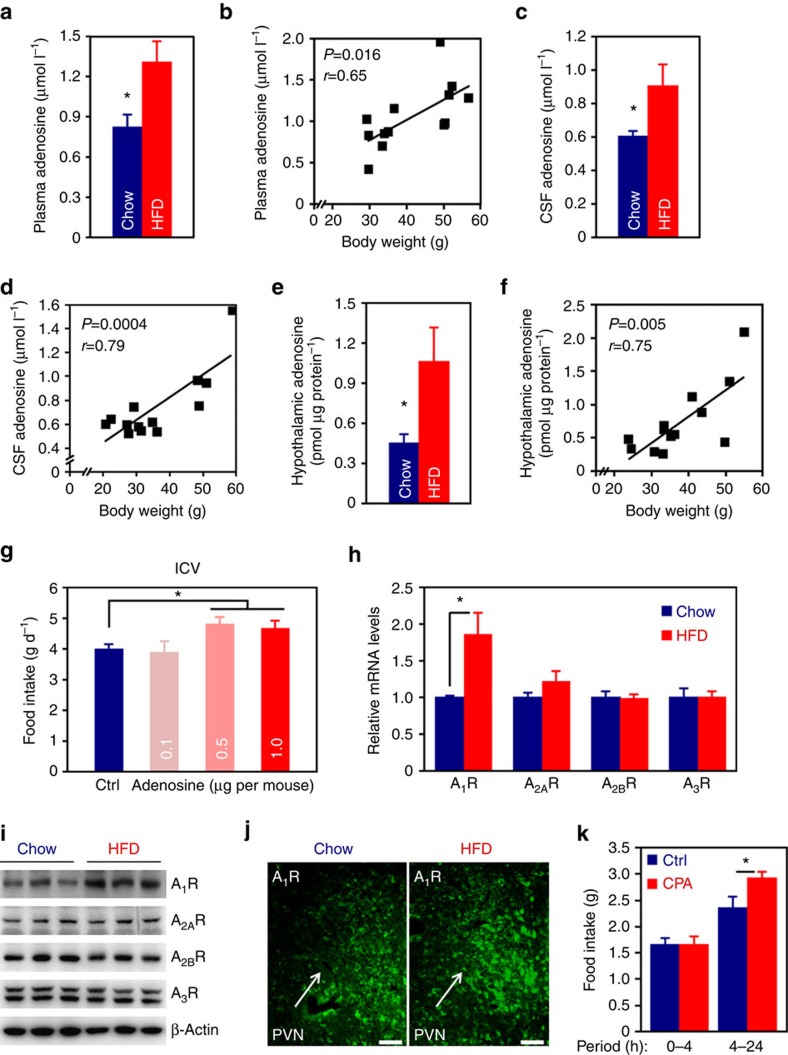
Aberration of the adenosine receptor signalling pathway in the hypothalamus of DIO mouse. (**a**) Plasma adenosine levels of chow- or 24 weeks HFD-fed mice. *n*=6 (Chow), 7 (HFD). (**b**) Correlation of plasma adenosine level with body weight, r, Pearson’s r; *P*, *P* value. (**c**) Adenosine levels in the CSF of chow- or 24 weeks HFD-fed mice. *n*=7. (**d**) Correlation of CSF adenosine level with body weight. (**e**) Hypothalamic adenosine contents of chow- or 24 weeks HFD-fed mice. *n*=7 (Chow), 6 (HFD). (**f**) Correlation of hypothalamic adenosine content with body weight. (**g**) Effect of i.c.v. administered adenosine on food intake. Ctrl, control. *n*=12 (Ctrl), 9 (0.1), 7 (0.5), 15 (1.0). (**h**) qRT-PCR analysis of the hypothalamic expression levels of adenosine receptors in chow- or HFD-fed mice. *n*=7 (Chow), 8 (HFD). (**i**) Western blot analysis of adenosine receptor expression in the hypothalami of chow- or HFD-fed mice. β-Actin was used as loading control. (**j**) Immunofluorescence staining of A_1_R in the PVN of hypothalamus of chow- or HFD-fed mouse. (**k**) Food intake of mice i.c.v. administered control or A_1_R agonist, CPA. *n*=9 (Ctrl), 7 (CPA). Data are presented as mean±s.e.m. *P<0.05, two-tailed Student’s *t*-test (**a**,**c**,**e**,**h**,**k**); one-way analysis of variance (ANOVA) with Bonferroni’s *post hoc* test (**g**).

Adenosine is a natural ligand for adenosine receptors. To examine whether there is any change of adenosine in the DIO animals, we fed male C57 BL/6 mice with a regular chow or high-fat diet (HFD). After 24 weeks of dietary treatment, we analysed the plasma adenosine level by using a sensitive fluorometric Elisa. The results showed that HFD feeding led to significantly elevated plasma adenosine level (Fig. 1a), and the plasma adenosine levels were well correlated with the body weights (Fig. 1b), suggesting adenosine metabolism was abnormal in the DIO mice. In addition, we measured the adenosine level in the cerebrospinal fluid (CSF) of 24 weeks HFD-fed mice. The CSF adenosine levels in these mice were evidently higher than the controls (Fig. 1c), and were also correlated with body weights (Fig. 1d). Moreover, when the hypothalamic adenosine was examined, we found that its contents in DIO mice were significantly elevated compared to chow-fed controls (Fig. 1e). Hypothalamic adenosine contents were also correlated to the body weights (Fig. 1f). To examine whether the change of brain adenosine occurs before the animal’s body weight is significantly increased, we measured the CSF levels in chow- or 2 weeks HFD-fed mice, at which time the animals’ body weights did not significantly differ (Chow, 21.50±0.88 g; HFD, 22.41±0.79 g. *P*=0.45, two-tailed Student’s *t*-test.). We found that the mean adenosine level was moderately, but significantly increased in HFD-fed animals (Supplementary Fig. 1a). Moreover, to exclude the effect of diet, we analysed the CSF adenosine levels in chow-fed *ob*/*ob* and wild-type mice. The result demonstrated that CSF level of adenosine was also elevated in the *ob*/*ob* mice (Supplementary Fig. 1b), indicating that alteration of brain adenosine is related to obesity, but not diet. Together, these results suggest that hypothalamic adenosine signalling might be involved in dietary obesity.

To examine the effect of intracerebroventricularly (i.c.v.) administered adenosine on food consumption, we implanted cannula directed to the third ventricle of C57 BL/6 mice. After the animals were fully recovered, we injected control artificial cerebrospinal fluid (aCSF) or adenosine to the brain. We found that adenosine at doses of 0.5 and 1.0 μg slightly, but significantly increased animal’s appetite (Fig. 1g).

### Overexpression of A_1_R in the PVN of DIO mice

Next, we examined the expression of adenosine receptors in the hypothalami of DIO mice. Quantitative reverse transcription PCR (qRT-PCR) result demonstrated that A_1_R, but not other subtypes, was significantly increased in the DIO mice (Fig. 1h). Western blot analysis further confirmed that A_1_R is overexpressed in the hypothalami of these animals (Fig. 1i; Supplementary Fig. 16). To verify these results, we did immunofluorescence staining on mouse brain sections, after confirming the specificity of A_1_R antibody (Supplementary Fig. 1c). Interestingly, we found that there was more aggregated fluorescence of A_1_R in the PVN of DIO mouse (Fig. 1j), indicating higher level of expression in this region. The immunofluorescence results did not reveal any significant change of A_1_R in other hypothalamic nuclei and extra-hypothalamic regions (Supplementary Fig. 2). We also examined the expression of A_2A_R, A_2B_R and A_3_R in the hypothalamus by immunofluorescence but did not notice any obvious changes (Supplementary Figs 3–5). Lastly, to test whether central A_1_R signalling is involved in the regulation of energy balance, we delivered N^6^-Cyclopentyladenosine (CPA), a selective agonist of A_1_R, to mouse brains by the i.c.v. route. Mice administered CPA consumed more chow foods than the controls (Fig. 1k), demonstrating that brain A_1_R regulates animal’s appetite.

### Overexpression of A_1_R in PVN neurons leads to obesity

**Figure 2:**
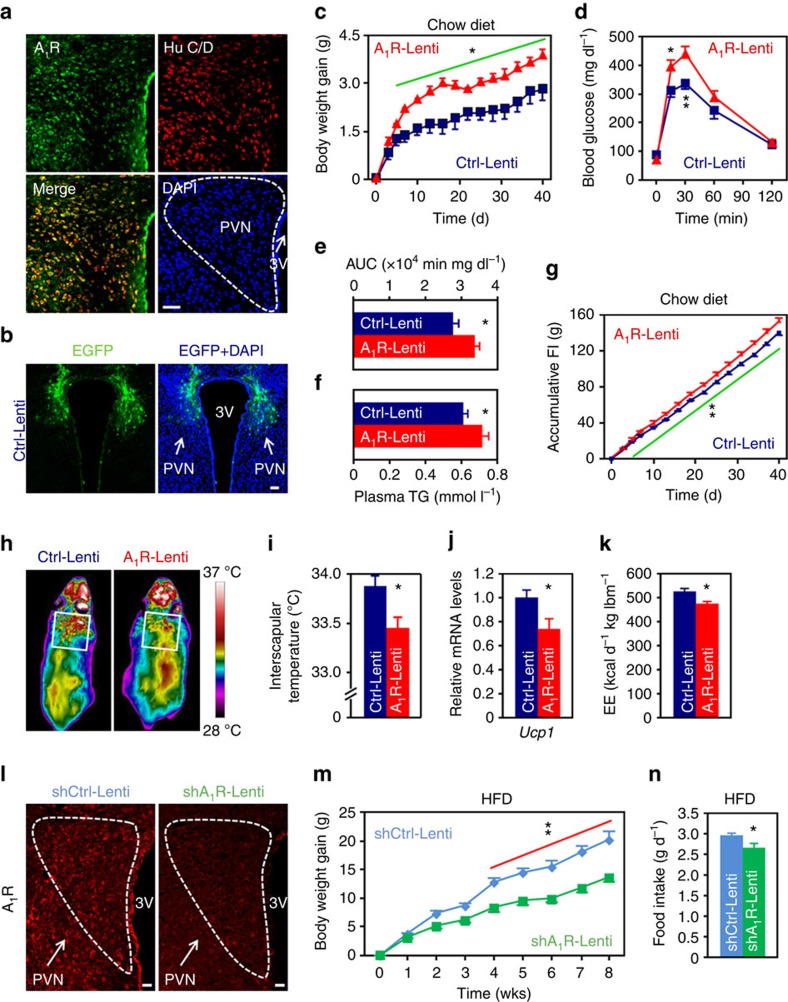
Effects of manipulations of A_1_R expression in PVN on systemic energy balance. (**a**) Double immunofluorescence staining of A_1_R (green) and neuronal marker Hu C/D (red) in mouse PVN. Cell nuclei were counterstained with DAPI (blue). 3V, third ventricle. Scale bar, 50 μm. (**b**) Expression of EGFP (green) after the injection of control lentivirus (Ctrl-Lenti) into PVN. Cell nuclei were counterstained with DAPI (blue). Scale bar, 50 μm. (**c**–**f**) Body weight gain (**c**), GTT (**d**), area under the curve (AUC) of GTT (**e**) and plasma triglycerides (TG) levels (**f**) were analysed. Mice were injected either Ctrl-Lenti or A_1_R-Lenti virus into the PVN. Ctrl-Lenti, *n*=6 (**c**,**f**), 7 (**d**,**e**). A_1_R-Lenti, *n*=6 (**f**), 7 (**c**–**e**). (**g**–**i**) Accumulative food intake (FI) (**g**), representative infrared images (**h**) and interscapular temperatures (**i**) of mice injected either Ctrl-Lenti or A_1_R-Lenti virus into the PVN. Ctrl-Lenti, *n*=6 (**g**), 7 (**i**). A_1_R-Lenti, *n*=7. (**j**) qRT-PCR analysis of the expression level of *Ucp1* in brown adipose tissue of mice injected Ctrl-Lenti (*n*=6) or A_1_R-Lenti (*n*=7) virus. (**k**) Daily energy expenditure (EE) of mice injected Ctrl-Lenti or A_1_R-Lenti virus. lbm, lean body mass. *n*=6. (**l**) Immunofluorescence images showing that A_1_R shRNA-expressing (shA1R-Lenti) lentivirus delivered into the PVN effectively reduced the expression of A_1_R in comparison with control (shCtrl-Lenti). 3V, third ventricle. Scale bar, 20 μm. (**m**,**n**) Body weight gain (**m**) and daily food intake (**n**) of mice injected either shCtrl-Lenti or shA_1_R-Lenti virus into the PVN. *n*=7. Data are presented as mean±s.e.m. *P<0.05, **P<0.01, two-tailed Student’s *t*-test (**e**,**f**,**i**–**k**,**n**); two-way analysis of variance (ANOVA) with Bonferroni’s *post hoc* test (**c**,**d**,**g**,**m**).

To study whether A_1_R is expressed in the neurons of PVN, we performed a double immunofluorescence staining on brain section. Indeed, we found A_1_R was expressed in most of the neurons (Fig. 2a), suggesting it may regulate energy balance via the action in these cells.

Next, we asked whether overexpression of A_1_R in PVN neurons would affect systemic energy balance. Besides, given a recent report showing that A_1_R in the Arc played a role in short-term regulation of food intake mainly by using chemogenetic approach^24^, we also included the Arc and DMH in our observation. We generated two lentiviral plasmids in which the expression of mouse A_1_R cDNA or enhanced green fluorescent protein (EGFP, as control) was driven by the neuron-specific *Synapsin* promoter^25^. These two plasmids were designated as A_1_R-Lenti or Ctrl-Lenti, respectively. We delivered the lentiviruses to mouse PVN, Arc or DMH by utilising a stereotaxic instrument. After confirming the success of surgery (Fig. 2b; Supplementary Figs 6 and 7a,d), we monitored the animal’s body weights and food intakes. We found that mice with ectopic expression of A_1_R in the PVN, but not Arc or DMH, gained more body weights than the controls (Fig. 2c, Supplementary Fig. 7b,e). Mice with overexpression of A_1_R in the PVN were intolerant to glucose challenge (Fig. 2d,e) and hypertriglyceridemic (Fig. 2f).

In addition, mice with overexpression of A_1_R in PVN were hyperphagic (Fig. 2g), whereas overexpression of A_1_R in Arc or DMH did not alter animal’s appetite (Supplementary Fig. 7c,f). Mice injected A_1_R-Lenti in the PVN had less wheel-running activities (Ctrl-Lenti, 6.22 ±0.40 km d^−1^; A_1_R-Lenti, 3.70±0.75 km d^−1^. *P*<0.05, two-tailed Student’s *t*-test.), lower interscapular temperature and reduced expression of *Ucp1* when compared to controls (Fig. 2h–j), suggesting the reduction of energy expenditure. Indeed, calorimetry analysis showed that these mice consumed significantly less O_2_ and produced less CO_2_ as well as heat than the controls (Fig. 2k; Supplementary Fig. 8a,b). We also extended our observation to arousal and anxiety-like behaviour. However, the data did not reveal any significant changes (Supplementary Fig. 8c–g).

We were also interested to know whether reduced expression of A_1_R in PVN would affect energy balance. To do this, we generated a lentiviral plasmid that expresses short hairpin RNA (shRNA) targeting mouse A_1_R (designated as shA_1_R-Lenti). After confirming the efficiency of knockdown (Fig. 2l), we delivered the shA_1_R-Lenti or control (shCtrl-Lenti) virus to mouse PVN. As expected, mice with reduced expression of A_1_R in PVN consumed significantly less foods and gained less body weights (Fig. 2m,n). Collectively, these data reveal an anabolism-promoting role of A_1_R expressed in the PVN neurons.

### Caffeine targets PVN neuronal A_1_R to regulate energy balance

**Figure 3:**
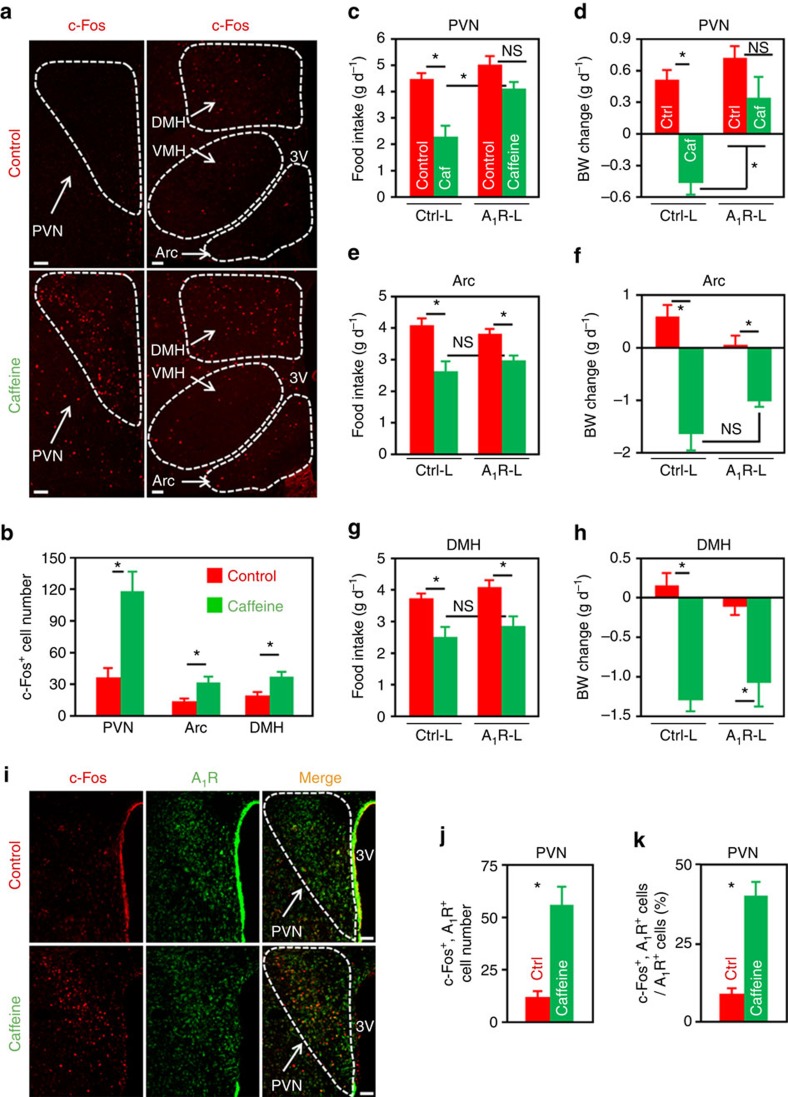
Overexpression of A_1_R in PVN neurons significantly attenuates caffeine’s effect on energy balance. (**a**) Immunofluorescence staining of c-Fos (red) in the paraventricular (PVN), arcuate (Arc), ventromedial (VMH) and dorsomedial (DMH) nuclei of mice infused with either caffeine (10 μg per mouse) or control. 3V, third ventricle. Scale bar, 50 μm. (**b**) The number of c-Fos^+^ cells in the PVN, Arc and DMH nuclei of control or caffeine administered mice. *n*=7. (**c**–**h**) Chow-fed mice were injected Ctrl-Lenti (Ctrl-L) or A_1_R-Lenti (A1R-L) virus into the PVN (**c**,**d**), Arc (**e**,**f**) or DMH (**g**,**h**). Meanwhile, cannula directed to third ventricle were implanted. The mice were then i.c.v. injected control or caffeine (10 μg per mouse), and 24- h food intake (**c**,**e**,**g**) and body weight change (**d**,**f**,**h**) were analysed. Ctrl, control; Caf, caffeine. For PVN, *n*=7; Arc, *n*=7 (Ctrl-L, Control), 6 (Ctrl-L, Caffeine), 5 (A_1_R-L); DMH, *n*=6 (Ctrl-L), 7 (A_1_R-L). (**i**) Double immunofluorescence staining of c-Fos (red) and A_1_R (green) in the PVN of control or caffeine administered mice. 3V, third ventricle. Scale bar, 50 μm. (**j**,**k**) The number of c-Fos^+^ and A_1_R^+^ cells (**j**), as well as the percentage of A_1_R^+^ cells expressing c-Fos (**k**) in the PVN of mice administered control (Ctrl) or caffeine. *n*=3. Data are presented as mean±s.e.m. *P<0.05, two-tailed Student’s *t*-test (**b**,**j**,**k**); one-way analysis of variance (ANOVA) with Bonferroni’s (**c**,**d**,**g**,**h**) or Newman–Keuls (**e**,**f**) *post hoc* test. NS, not significant.

Given that hypothalamic A_1_R is involved in energy balance (Figs 1 and 2), next, we asked whether caffeine, the antagonist of adenosine receptors, would directly regulate the activities of hypothalamic neurons. Initially, we found that i.c.v. administration of caffeine at doses ≥10 μg per mouse significantly reduced animal’s appetite (Supplementary Fig. 9a). Hence, we chose the dose of 10 μg per mouse whenever caffeine is administered via the i.c.v. route, unless otherwise noted. Administration of caffeine into mouse brain significantly increased the numbers of c-Fos^+^ cells in the PVN, Arc and DMH nuclei (Fig. 3a,b), indicating that caffeine stimulates the activities of neurons in the hypothalamic nuclei involved in energy balance control.

The aforementioned results led us to ask whether caffeine modulates energy balance through its action on hypothalamic A_1_R. To do this, we injected Ctrl-Lenti or A_1_R-Lenti virus into the PVN, Arc or DMH nucleus. In addition, the animals were implanted cannula directed toward third ventricles, and allowed to fully recover from surgeries. We then injected aCSF or 10 μg of caffeine into the brain. Intriguingly, overexpression of A_1_R in PVN, but not Arc or DMH, significantly abolished caffeine’s effects on appetite (Fig. 3c,e,g) and body weight balance (Fig. 3d,f,h), demonstrating that PVN is a critical site for caffeine to regulate energy balance.

Since A_1_R is mainly coupled to G_i/o_ protein, its antagonism will evoke the activity of neurons. Hence, central administration of caffeine would excite A_1_R^+^ neurons in the PVN. To test this prediction, we i.c.v. administered control or caffeine into mouse brains. After double immunostaining by using antibodies against A_1_R and c-Fos, we found that caffeine readily excites A_1_R^+^ cells in the PVN, shown by the greater number of cells expressing both c-Fos and A_1_R (Fig. 3i,j), and the increased ratio of double positive cells among A_1_R^+^ cells (Fig. 3k).

### Brain administration of caffeine ameliorates dietary obesity

**Figure 4:**
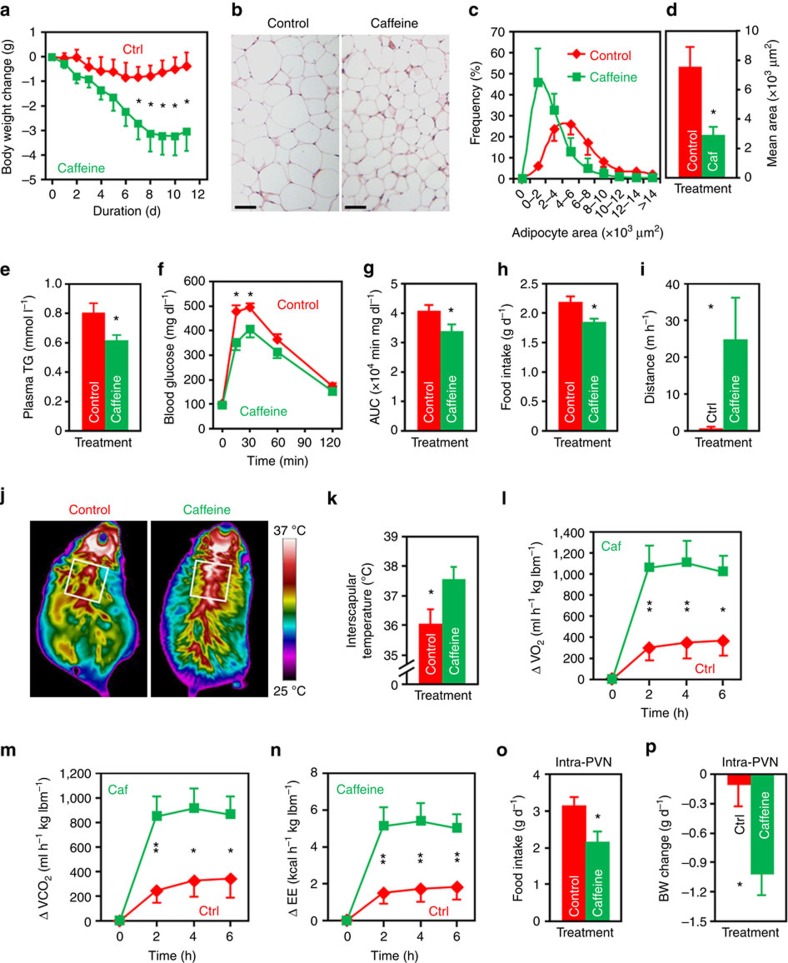
Central administration of caffeine reduces the body weights and improves obesity-related syndrome in DIO mice. (**a**) Daily i.c.v. administration of caffeine (10 μg per mouse) significantly reduced the body weights of DIO mice. Ctrl, aCSF injected mice. *n*=9 (Ctrl), 11 (Caffeine). (**b**–**d**) H&E staining (**b**), distribution of area (based on 100 cells per mouse) (**c**), mean area (**d**) of adipocytes of epididymal white adipose tissue (eWAT) from mice administered control or caffeine (Caf). *n*=3. (**e**–**g**) Post-treatment plasma triglycerides (TG) levels (**e**), GTT (**f**) and the AUC of GTT (**g**) of mice injected control or caffeine. *n*=6 (**e**). *n*=7 (Control), 9 (Caffeine) (**f**,**g**). (**h**) Food intake of mice i.c.v. injected control or caffeine. *n*=9 (Control), 11 (Caffeine). (**i**) Distance travelled during the first hour by mice i.c.v. infused control or caffeine. *n*=6 (Ctrl), 5 (Caffeine). (**j**) Representative infrared images acquired 4 h post i.c.v. injection. (**k**) Quantification of the highest 10% temperatures in the interscapular area. *n*=4 (Control), 5 (Caffeine). (**l**–**n**) Changes of O_2_ consumption (**l**), CO_2_ production (**m**) and energy expenditure (EE) (**n**) of the DIO mice immediately after the i.c.v. injection of control or of caffeine. lbm, lean body mass. *n*=8. (**o**,**p**) Twenty-four hours food intake (**o**) and body weight change (**p**) of mice administered control or caffeine (1 μg per mouse) into the PVN. *n*=9. Data are presented as mean±s.e.m. *P<0.05, **P<0.01, two-tailed Student’s *t*-test, comparison between caffeine and control groups (**d**,**e**,**g**–**i**,**k**,**o**,**p**); two-way analysis of variance (ANOVA) with Bonferroni’s *post hoc* test (**a**,**f**,**l**–**n**).

Given that A_1_R is overexpressed in the PVN of DIO mice (Fig. 1h–j), and PVN is the key hypothalamic region for caffeine to regulate energy metabolism (Fig. 3c–h), it seems intuitive that caffeine might counteract obesity through its action in the PVN. To test this, we performed third ventricle cannulation surgery on HFD-fed mice. After that, we i.c.v. injected aCSF or 10 μg of caffeine to these animals on a daily basis. Mice administered caffeine in the brain gained significantly less body weights than the controls on day 7 of the treatment and thereafter (Fig. 4a). The adipocyte sizes of epididymal white adipose tissue were much smaller than the controls (Fig. 4b–d). In addition, plasma triglycerides (TG) levels of caffeine-infused mice were lower than the controls (Fig. 4e). Glucose tolerance of these mice was also improved (Fig. 4f,g).

To elucidate the causes of caffeine-related reduction of dietary obesity, we measured both energy intake and expenditure. We found that mice given caffeine consumed significantly less HFD (Fig. 4h) but had more spontaneous wheel-running activities (Fig. 4i). Mice administered caffeine immediately before the dark cycle tended to have shorter duration of food intake, but did not spend more time on non-food intake-related locomotor activities (Supplementary Fig. 9b,c), indicating that the increasing of spontaneous locomotor activity (Fig. 4i) did not directly contribute to the reduction of food intake (Fig. 4h). In addition, after caffeine treatment, the insterscapular temperature and expression levels of thermogenesis-promoting genes in the brown adipose tissue were significantly elevated (Fig. 4j,k; Supplementary Fig. 9d). Indirect calorimetry analysis showed that brain administration of caffeine promoted the consumption of O_2_, and the production of CO_2_ as well as heat (Fig. 4l–n). To examine the effect of 11-day caffeine treatment on the expression of adenosine receptors, we performed western blot analysis on hypothalamic samples. The result did not show any significant change between the two treatment groups (Supplementary Fig. 9e).

Since PVN is the major site for A_1_R and caffeine to regulate energy balance (Figs 2 and 3), we were interested to know whether injection of caffeine directly to this nucleus would affect appetite and body weight balance. Caffeine administered at ≤0.5 μg per mouse did not show a significant effect (food intake: control, 3.11±0.26 g d^−1^; caffeine (0.5 μg), 2.50±0.40 g d^−1^. *P*=0.18, two-tailed Student’s *t*-test). However, the food intakes and body weights of mice given caffeine at the dose of 1 μg were significantly reduced in comparison with the controls (Fig. 4o,p), further confirming PVN is the key brain region for caffeine to counteract obesity.

We also analysed the effect of i.c.v. administered caffeine on anxiety-like behaviour and arousal. In the open field test, mice acutely given caffeine tended to stay longer in the central region (*P*=0.07, Supplementary Fig. 9f), suggesting an anxiolytic function of caffeine. We did not notice any significant changes in the elevated plus maze and light/dark box tests (Supplementary Fig. 9g,h). It is well recognized that caffeine is a psychoactive agent that promotes wakefulness^26^. However, considering that the main purpose of our study is to investigate the roles of caffeine and hypothalamic adenosine receptor in energy balance, we performed most of the injections of reagents immediately before the onset of dark cycle, because for C57 BL/6 mice, 67–83% of the amount of food was consumed during this period^27^. In contrast, studies focusing on arousal were mostly conducted during the light cycle, mainly because mouse is a nocturnal species. Nonetheless, i.c.v. administration of caffeine immediately before the dark cycle did not alter the wakefulness time during the first 4 h of the following light cycle (Supplementary Fig. 9i,j), demonstrating that caffeine administered at this time point did not affect the sleep/wakefulness homeostasis. Taken together, these data demonstrate that central caffeine treatment reduces the body weights and improves obesity-related symptoms in the DIO mice.

### Effect of peripheral caffeine treatment on energy balance

**Figure 5:**
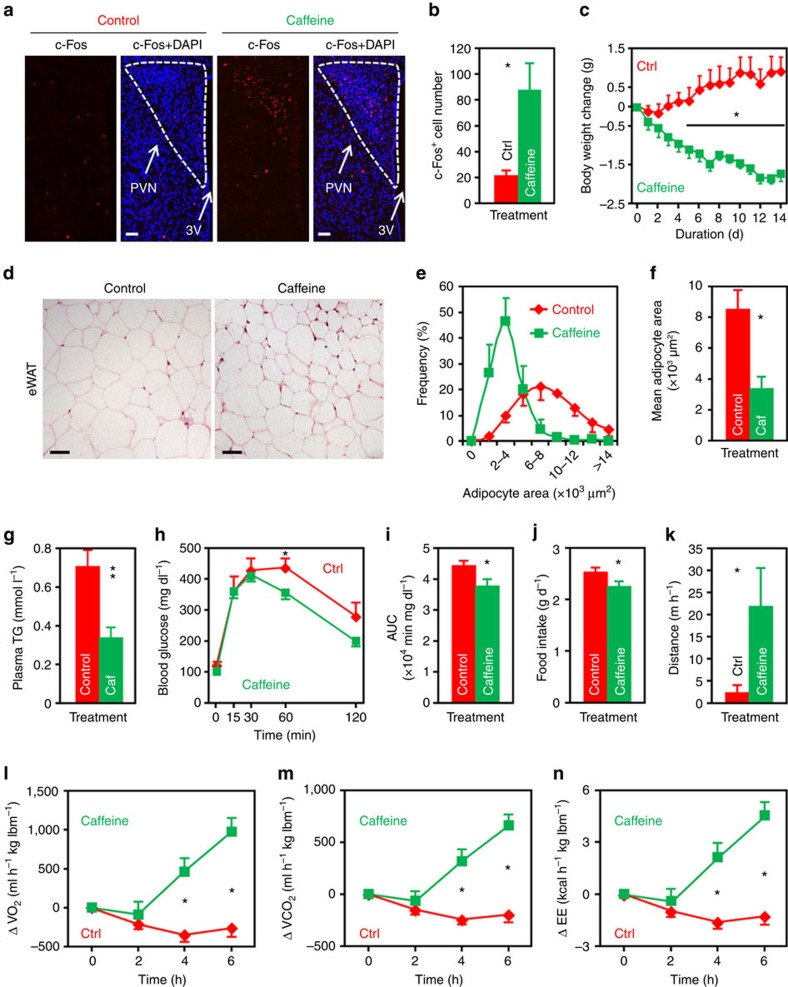
Peripheral caffeine treatment ameliorates diet-induced obesity. (**a**) Peripheral caffeine treatment elicits neuronal activities in the PVN. Immunofluorescence staining of c-Fos (red) in the PVN of mice administered control saline or caffeine (60 mg kg^−1^) by using oral gavage. Cell nuclei were counterstained with DAPI. 3V, third ventricle. Scale bar, 50 μm. (**b**) Numbers of c-Fos^+^ cells in the PVN. *n*=7 (Ctrl), 6 (Caffeine). (**c**) Body weight changes of DIO mice administered control (Ctrl) or caffeine (60 mg kg^−1^) by using oral gavage. *n*=7. (**d**–**f**) H&E staining (**d**), distribution of area (based on 100 cells per mouse) (**e**), and the mean area (**f**) of adipocyte of epididymal white adipose tissue (eWAT) from control or caffeine (Caf) injected mice. Scale bar, 50 μm. *n*=4. (**g**–**i**) Post-treatment plasma triglycerides (TG) levels (**g**), GTT (**h**) and the AUC of GTT (**i**) of mice injected control or caffeine. Control, *n*=7 (**g**), 10 (**h**,**i**). Caffeine, *n*=8 (**g**), 10 (**h**,**i**). (**j**) Daily food intake of control or caffeine administered mice. *n*=7. (**k**) Distance travelled in the first hour by mice administered control of caffeine. *n*=8. (**l**–**n**) Changes of O_2_ consumption (**l**), CO_2_ production (**m**) and energy expenditure (EE) (**n**) of the DIO mice immediately after the administration of control or caffeine. lbm, lean body mass. *n*=8. Data are presented as mean±s.e.m. *P<0.05, **P<0.01, two-tailed Student’s *t*-test (**b**,**f**,**g**,**i**–**k**); two-way analysis of variance (ANOVA) with Bonferroni’s *post hoc* test (**c**,**h**,**l**–**n**).

Given that caffeine is mostly consumed by the oral route in the human population, next, we asked whether peripheral administration of caffeine would also ameliorate dietary obesity. To begin with, we examined the dose-response effect of peripherally administered caffeine on food intake. Caffeine administered at doses ≥60 mg kg^−1^ by oral gavage significantly suppressed the appetite of DIO mice (Supplementary Fig. 10a), so we chose the lowest effective dose, that is, 60 mg kg^−1^ in the present study. Intragastrical infusion of caffeine evidently increased the numbers of c-Fos^+^ cells in the PVN (Fig. 5a,b), Arc and DMH nuclei (Supplementary Fig. 10b,c), suggesting that peripherally injected caffeine might regulate energy metabolism through the modulation of hypothalamic, in particular PVN, neuronal activities.

To study the effect of peripherally administered caffeine on energy balance, we injected saline or caffeine (60 mg kg^−1^) to DIO mice by oral gavage. Two-week caffeine treatment significantly reduced the body weights of DIO mice (Fig. 5c). Adipocytes of caffeine-treated mice were much smaller in size (Fig. 5d–f). Plasma TG level was markedly decreased (Fig. 5g), and glucose tolerance was improved (Fig. 5h,i). To interrogate the causes of peripheral caffeine treatment-induced body weight reduction, we measured the food intake and energy expenditure. We found that peripherally administered caffeine significantly reduced the food intakes (Fig. 5j), and increased the wheel-running activities of DIO mice (Fig. 5k). Mice receiving caffeine consumed more O_2_, and produced more CO_2_ as well as heat (Fig. 5l–n). We also analysed the expression of adenosine receptors in the hypothalami of 2-week caffeine or saline-treated mice by using western blot, but did not find any significant change (Supplementary Fig. 10d). Together, the results demonstrate that peripheral caffeine treatment ameliorates obesity through both the reduction of food intake and the promotion of energy expenditure.

### Blocking Oxt attenuates caffeine’s effect on energy balance

**Figure 6:**
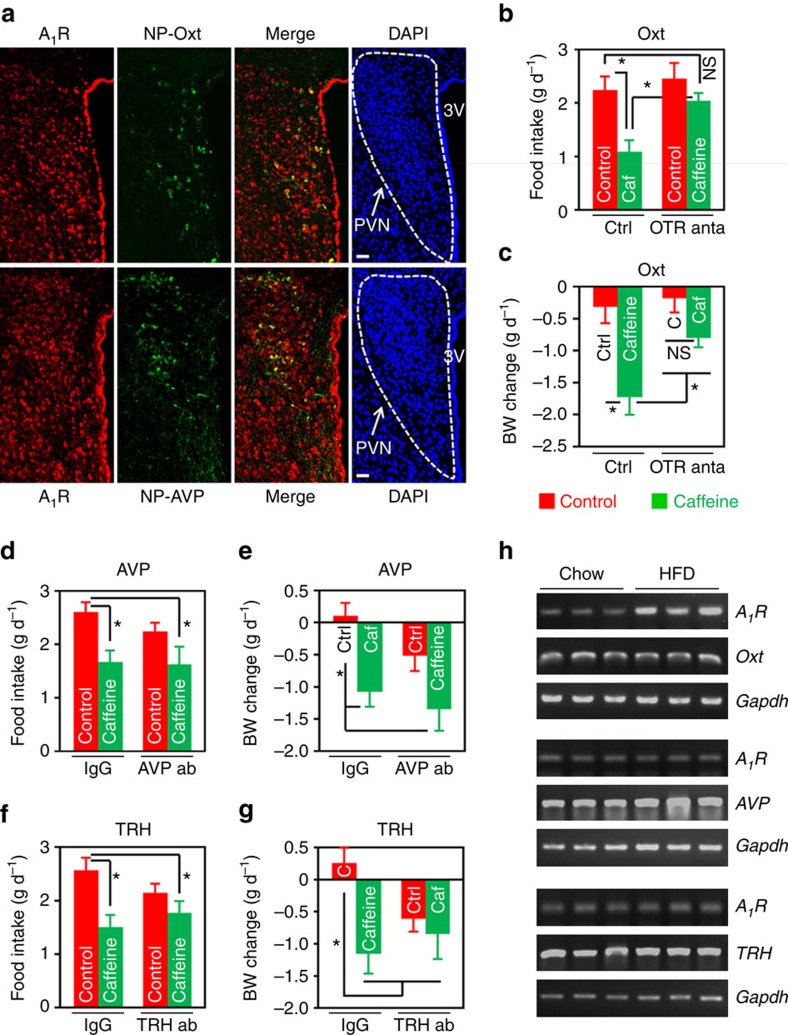
Oxt mediates caffeine's effect on energy balance in the DIO mice. (**a**) Double immunofluorescence staining of A_1_R (red) and Neurophysin I (NP-Oxt, green) or Neurophysin II (NP-AVP, green), which is co-expressed with Oxt or AVP in the PVN, respectively. Cell nuclei were counterstained with DAPI (blue). 3V, third ventricle. Scale bar, 50 μm. (**b**–**g**) HFD-fed mice were i.c.v. administered aCSF or IgG as control (Ctrl), and 2 μg of Oxt receptor (OTR) antagonist (anta), L-368,899 (**b**,**c**), or 0.5 μg of antibody against AVP (**d**,**e**) or TRH (**f**,**g**). An hour later, mice were i.c.v. injected control or 10 μg of caffeine (Caf or C). Twenty-four hours food intake (**b**,**d**,**f**) and body weight change (**c**,**e**,**g**) were then measured. ab, antibody. In OTR antagonist experiment, *n*=10 (Ctrl+Ctrl), 11 (Ctrl+Caf), 9 (OTR anta+Ctrl), 13 (OTR anta+Caf); AVP antibody, *n*=11 (IgG+Ctrl), 9 (IgG+Caf), 7 (AVP ab+Ctrl), 14 (AVP ab+Caf); TRH antibody, *n*=7 (IgG+Ctrl), 7 (IgG+Caf), 8 (TRH ab+Ctrl), 10 (TRH ab+Caf). (**h**) Single-cell RT-PCR analysis of A_1_R expression in Oxt, AVP or TRH-expressing cells isolated from the PVN of chow or HFD-fed mice. Gapdh was used as an internal control. Data are presented as mean±s.e.m. *P<0.05, one-way analysis of variance (ANOVA) with Bonferroni’s (**b**,**d**,**e**,**f**) or Newman–Keuls (**c**,**g**) *post hoc* test. NS, not significant.

We have shown that caffeine regulates energy balance mainly through its action on A_1_R in the PVN (Fig. 3c–h). It is well recognized that in the PVN there are several types of peptidergic neurons, such as Oxt, AVP, TRH and corticotropin-releasing hormone (CRH)^12^,^18^,^28^. Next, we investigated the type(s) of neurons that express A_1_R in the PVN by double immunofluorescence staining with antibodies against A_1_R and Neurophysin I (NP-Oxt), Neurophysin II (NP-AVP), TRH or CRH. NP-Oxt and NP-AVP are carrier proteins that are specifically associated with Oxt (Supplementary Fig. 11a) or AVP, respectively^29^,^30^. The data demonstrated that A_1_R was expressed in both the Oxt (NP-Oxt^+^) and AVP (NP-AVP^+^) neurons (Fig. 6a). A_1_R was also expressed in very few TRH, but not CRH neurons (Supplementary Fig. 11b,c).

To identify the PVN neuropeptide(s) that mediate caffeine’s effects on energy balance, we performed the following pharmacological study. We i.c.v. administered Oxt receptor (OTR) antagonist, L-368,899, or antibodies against AVP or TRH to mouse brains. An hour later, aCSF or 10 μg of caffeine was infused into mouse brains via the same route. The results demonstrated that pre-treatment with Oxt receptor antagonist L-368,899, but not AVP or TRH antibody, significantly attenuated caffeine’s effects on food intake and body weight (Fig. 6b–g), indicating Oxt is the key mediator of caffeine’s effect on energy metabolism.

We were also interested to find out the type(s) of neurons in which the expression of A_1_R was elevated in the DIO mice (Fig. 1j). To do this, we employed single-cell RT-PCR to examine the messenger RNA levels of A_1_R in specific neurons isolated from PVN. Interestingly, the messenger RNA level of A_1_R was significantly elevated in the Oxt, but not the other two types of neurons (Fig. 6h), suggesting A_1_R might play a role in DIO through its action in the Oxt neuron.

### Oxt neuron-specific knockdown of A_1_R mitigates DIO

**Figure 7:**
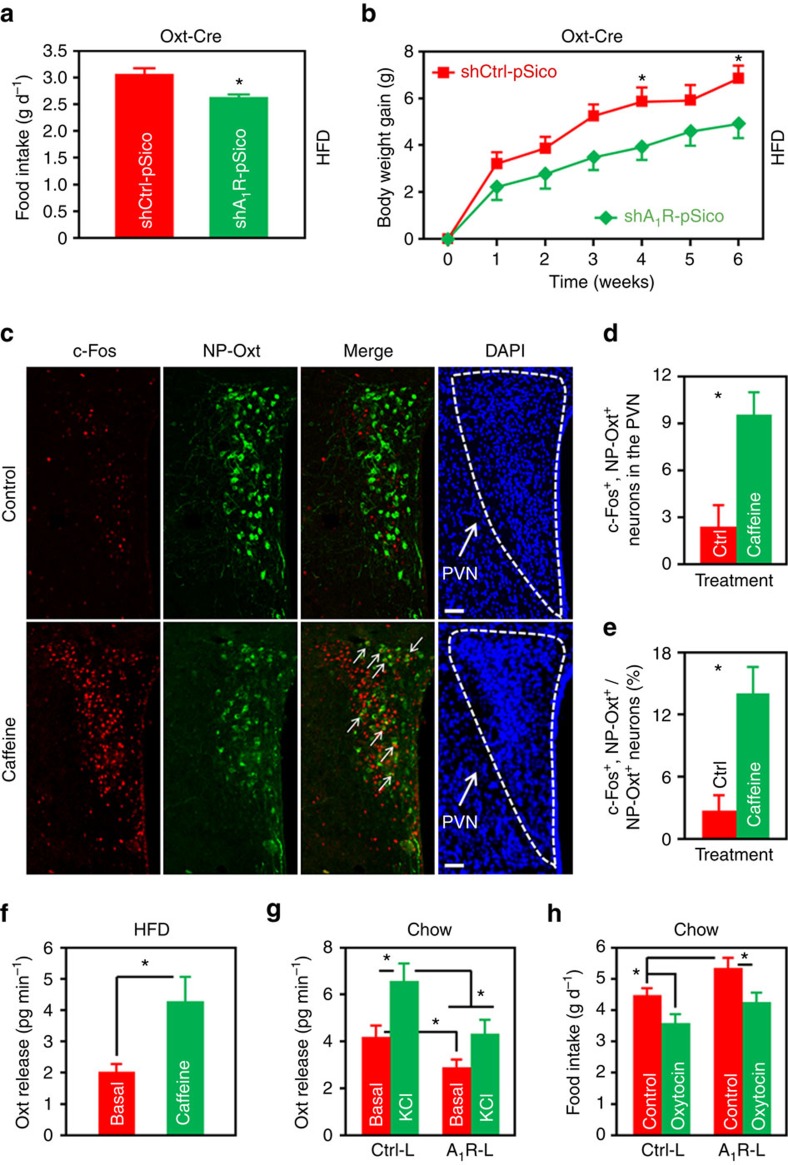
Caffeine and A_1_R regulate the PVN Oxt release. (**a**,**b**) HFD intake (**a**) and body weight gain (**b**) of Oxt-Cre mice injected either shCtrl-pSico or shA_1_R-pSico lentivirus into the PVN. *n*=6 (shCtrl-pSico), 7 (shA_1_R-pSico). (**c**) Double immunofluorescence staining of c-Fos (red) and NP-Oxt (green) in the PVN of mice i.c.v. administered control or caffeine (10 μg per mouse). Cell nuclei were counterstained with DAPI (blue). Arrows indicate c-Fos and NP-Oxt co-expressing cells. Scale bar, 50 μm. (**d**,**e**) Number of c-Fos^+^ and NP-Oxt^+^ cells (**d**), as well as the percentage of NP-Oxt^+^ cells expressing c-Fos in the PVN (**e**). *n*=3 (Ctrl), 4 (Caffeine). (**f**) PVN slices of 12 weeks HFD-fed mice were dissected from the brains. Basal and caffeine (2 mmol l^−1^) elicited Oxt release were measured. *n*=8. (**g**) Chow-fed mice were injected Ctrl-Lenti (Ctrl-L) or A_1_R-Lenti (A1R-L) virus into the PVN. The animals were allowed to recover from surgeries, and then spontaneous (Basal) and high K^+^ (KCl) elicited Oxt release of PVN slices were examined. *n*=6 (Ctrl-L), 8 (A_1_R-L). (**h**) Chow-fed mice were injected Ctrl-L or A_1_R-L virus into the PVN, and cannulas directed to third ventricle were implanted. The mice were then i.c.v. administered control or 1 μg of Oxt, and food intake was measured. *n*=7 (Control), 6 (Oxytocin). Data are presented as mean±s.e.m. *P<0.05, two-tailed Student’s *t*-test (**a**,**d**,**e**,**f**); two-way analysis of variance (ANOVA) with Bonferroni’s *post hoc* test (**b**); or one-way ANOVA with Newman–Keuls *post hoc* test (**g**,**h**).

To further explore the Oxt neuron-specific role of A_1_R in DIO, we constructed a Cre-inducible, A_1_R-targeted shRNA lentiviral expression plasmid based on the pSico vector (Supplementary Fig. 12a)^31^. We designated this and the control plasmids as shA_1_R-pSico and shCtrl-pSico, respectively. Double immunofluorescence staining revealed that, after the transduction of shA_1_R-pSico lentivirus, expression of A_1_R in PVN Oxt neurons reduced ∼40% (Supplementary Fig. 12b,c). We then injected the shA_1_R-pSico or control lentivirus into the PVN of Oxt-Cre mice^32^, respectively. The results showed that Oxt neuron-specific knockdown of A_1_R significantly reduced the food intakes and body weight gains of mice under HFD treatment (Fig. 7a,b).

### Effect of caffeine or A_1_R on Oxt release from the PVN

Previously, we have shown that impaired Oxt release from PVN is involved in the pathogenesis of DIO^18^. Next, we asked whether caffeine or A_1_R would regulate the releasing of Oxt. We first examined the Oxt neuron activity after the mice had been i.c.v. administered caffeine or aCSF. We were able to find that caffeine greatly stimulated the activities of these neurons in the PVN (Fig. 7c–e), suggesting that it may evoke Oxt release. We then performed an Oxt release assay to examine this possibility. When PVN slices dissected from DIO mice were treated with caffeine (2 mmol l^−1^), the *ex vivo* Oxt releasing rate was significantly increased (Fig. 7f). Indeed, caffeine seemed to augment Oxt-induced OTR signalling in the mouse brain (Supplementary Fig. 13), although A_1_R did not interact directly with OTR (Supplementary Fig. 14). Moreover, when we induced overexpression of A_1_R in the PVN of chow-fed mice by the injection of A_1_R-Lenti virus, the rates of spontaneous and high potassium evoked Oxt release were significantly attenuated (Fig. 7g). The modulation of Oxt release by A_1_R does not seem to be a direct effect, because double immunofluorescence staining of NP-Oxt and A_1_R did not reveal any significant co-localization in the nucleus of solitary tract (Supplementary Fig. 15), a region heavily innervated by Oxt neurons.

Lastly, we interrogated the role of brain Oxt in A_1_R-mediated regulation of energy balance. To do this, we injected A_1_R-Lenti or Ctrl-Lenti virus to the PVN, and implanted cannulas directed to the third ventricle of chow-fed mice. After the animals were recovered from surgeries, we administered control or Oxt (1 μg per mouse) into the brain. Consistent with our previous result (Fig. 2g), mice consumed more foods when A_1_R was overexpressed in the PVN neurons. However, treatment with Oxt significantly abolished this effect (Fig. 7h), demonstrating that it indeed antagonizes A_1_R’s effect on energy balance.

## Discussion

Coffee consumption has been linked to better metabolic outcomes^4^,^33^, however, the underlying mechanisms remain largely unknown. Caffeine is one of the major active ingredients in coffee, tea and soft drinks, and hypothalamus in the brain plays a fundamental role in controlling animal’s energy homeostasis^11^,^12^. Here, we found that adenosine levels were increased in the plasma, CSF and hypothalamus (Fig. 1a–f), and A_1_R was overexpressed in the PVN Oxt neurons of DIO mice (Fig. 6h). Ectopic expression of A_1_R in PVN neurons enhanced appetite and promoted body weight gain (Fig. 2c,g). Central or peripheral administration of caffeine negatively regulated energy balance in the DIO mice, demonstrated by the reduction of food intake and body weight, as well as the increasing of energy expenditure (Figs 4 and 5). In both treatments, administration of caffeine improved the glucose tolerance and decreased the plasma TG levels in DIO mice (Figs 4 and 5). Moreover, we found that caffeine targets the A_1_R expressed in PVN Oxt neurons to regulate energy metabolism (Figs 6 and 7; Supplementary Fig. 11). Collectively, our results demonstrate that central caffeine treatment negatively regulates energy balance by promoting the release of Oxt.

Caffeine is a CNS stimulant and antagonist of adenosine receptors. To date, four subtypes of adenosine receptors have been identified in mammals^5^. Characterization of the expression pattern showed that adenosine receptors are widely distributed in the body^5^. Within the CNS, both A_1_R and A_2A_R show high levels of expression^5^, albeit that the A_2A_R is enriched in the basal ganglia, whereas A_1_R is widely distributed on neurons^5^,^7^. In this study, we found that A_1_R is expressed in the hypothalamic nuclei regulating energy balance (Figs 1j and 2a), suggesting that it may regulate systemic energy balance via actions in the hypothalamus. We examined the four subtypes of adenosine receptors by utilising qRT-PCR, Western blot and immunofluorescence. Interestingly, we found that only A_1_R showed elevated expression in the PVN of DIO mice (Fig. 1h–j; Supplementary Figs 2–5). Moreover, caffeine-evoked excitation of a significant portion of A_1_R^+^ cells in the PVN (Fig. 3i–k), which is in agreement with the neuronal inhibitory function of this receptor. Indeed, previous studies displayed that agonist-mediated activation of A_1_R decreased the neuronal activities in the dorsal root ganglia^34^, lateral horn^35^, entorhinal cortex^36^, whereas caffeine could facilitate the release of glutamate from neurons in the cerebral cortex^37^. Furthermore, overexpression of A_1_R in the PVN, but not Arc or DMH neurons significantly attenuated caffeine’s effect on energy metabolism (Fig. 3c–h), indicating that A_1_R expressed in the PVN is a critical target for caffeine to control energy balance. Together, our results demonstrate that the anti-obesity effect of centrally administered caffeine is primarily mediated by antagonizing A_1_R expressed in PVN neurons.

A recent study reported that adenosine released from astrocytes in the mediobasal hypothalamus (MBH) inhibits food intake of mice^24^. The authors found that in mice fed a regular chow diet, acute activation of MBH astrocytes by using chemogenetic approach suppresses the firing rates of Agrp neurons. The inhibitory function of adenosine/A_1_R is consistent with our result showing that A_1_R plays an inhibitory role in neuropeptide exocytosis (Fig. 7g). However, it seems unlikely that the inhibition of Agrp neurons by astrocyte-released adenosine could play a role in dietary obesity, because under HFD feeding, mice tend to accumulate astrocytes in the MBH^38^. Hence, the physiological and pathological significance of such a regulatory system is not yet clear.

In the present study, other than A_1_R, we did not find any significant change of the hypothalamic expression of adenosine receptors between the DIO and chow-fed mice (Fig. 1h,i; Supplementary Figs 3–5). Previously, it had been shown that striatal A_2A_R plays a critical role in habit formation^39^,^40^,^41^, and stimulation of A_2A_R reduces the affinity of agonist binding to dopamine receptor D_2_ (ref. 42). Given that downregulation of striatal D_2_R is required in addiction-like reward dysfunction and compulsive eating in obese rats^43^, it is conceivable that A_2A_R expressed in the striatum might be involved in the pathogenesis of DIO. Antagonism of A_2A_R by caffeine would presumably increase the activity of D_2_R, and consequently alleviate the food addiction of DIO animals.

Neuropeptides are a subfamily of neurotransmitter playing a pivotal role in the regulation of energy homeostasis^11^,^12^. Dysregulation of hypothalamic neuropeptides at various levels is linked to obesogenesis^18^,^44^. Since we found that PVN is the main brain region for caffeine to regulate energy metabolism (Fig. 3c–h), we focused on this particular structure, aiming to elaborate the mechanism of caffeine-A_1_R interaction on energy metabolism. Our data showed that A_1_R was expressed in the Oxt and AVP neurons of PVN (Fig. 6a), and administration of caffeine led to the excitation of Oxt neurons (Fig. 7c–e). Moreover, the results of two complementary experiments show that the caffeine-A_1_R signalling system does use Oxt to suppress appetite and reduce body weight (Figs 6b–g and 7h), which is consistent with the anorexic effect of Oxt (refs 18, 27). Interestingly, previous studies have shown that A_1_R played a crucial role in the regulation of peptide and hormone release. For example, activation of A_1_R by selective agonist, 2-chloroadenosine, suppressed KCl evoked release of Dynorphin A(1-8) from the hippocampal synaptosomes^45^. Endogenous adenosine was able to cause tonic inhibition of transient Ca^2+^ current and evoked exocytosis in neurohypophysial terminals^46^. In addition, stimulation of A_1_R in the spinal cord inhibits the exocytosis of calcitonin gene-related peptide^47^. These studies unequivocally showed that A_1_R inhibits the release of neuropeptides and hormones. Taken together, the evidences demonstrate that Oxt is a critical mediator of caffeine-induced negative regulation of energy balance in the DIO mice.

There are few available medications for the long-term treatment of obesity^3^. Efficacy and safety are two major concerns over anti-obesity medications. Caffeine, however, has been deemed safe when consumed up to 400 mg d^−1^ for the general population^48^. In agreement with our observations (Figs 4j–n and 5l–n), both the energy expenditure and skin temperature of human subjects administered caffeine were increased compared to controls^49^,^50^. Moreover, the combinatorial modality of caffeine and ephedrine, the principal active ingredient in the Chinese herbal plant Ma Huang, was once widely used as a dietary supplement to reduce body weight. The adverse effect associated with ephedrine led to the cessation of using it as an anti-obesity supplement^51^. However, given that caffeine negatively regulates energy balance, caffeine or its derivatives might be interesting candidate agents to combat obesity.

In the present study, we found that central or peripheral administration of caffeine improved the glucose tolerance of DIO mice (Figs 4f,g and 5h,i). This is in agreement with previous studies conducted in human populations showing that consumption of coffee reduces the risk of type 2 diabetes mellitus^52^,^53^. With regard to the mechanisms, we show that caffeine excites Oxt neurons (Fig. 7c–e) and stimulates the release of Oxt from PVN (Fig. 7f). Given that Oxt treatment improved the glucose tolerance in DIO animals^27^,^54^, it is likely that caffeine modulates glucose metabolism via its action on Oxt neurons. It should be noted that peripherally expressed adenosine receptors were shown to be involved in the regulation of glucose homeostasis. For example, selective antagonism of A_2B_R increased glucose infusion rate and uptake into skeletal muscle as well as brown adipose tissue^55^. In contrast, other studies employing whole-body knockout mice showed that A_2B_R is required to maintain normal glucose level and insulin sensitivity^56^,^57^. Clearly, adenosine receptors play a pivotal role in animal’s glucose homeostasis, although the tissue specific functions need to be further elucidated.

Caffeine plays a prominent role in fatty acid metabolism. It was initially discovered that theophylline elicits the release of glycerol and free fatty acids from adipose tissue^58^. Moreover, A_1_R is expressed in adipose tissue and its activation inhibits lipolysis. Thus, peripherally administered caffeine might promote lipolysis by directly inhibiting A_1_R expressed in adipocytes. The role of adenosine in brown adipose tissue lipolysis is more complexed^59^,^60^,^61^. In the present study, we found that brain administration of caffeine reduced the TG levels in DIO mice (Fig. 4e), and caffeine stimulates the activities of Oxt neurons (Fig. 7c–e). Given that one study found that Oxt neurons are part of the sympathetic nervous system outflow from brain to white adipose tissue (ref. 62), caffeine might employ the brain-fat axis to regulate lipid metabolism.

In summary, we found that aberrations of the adenosine-A_1_R signalling pathway occur in the hypothalamus of DIO mouse. A_1_R expressed in PVN Oxt neurons plays a critical role in the regulation of energy homeostasis. Administration of caffeine by central or peripheral route suppresses appetite, increases energy expenditure, and reduces the body weight of DIO mice. PVN Oxt is a critical mediator of the anti-obesity effect of caffeine. Hence, targeting PVN A_1_R by caffeine or its derivatives could represent a relevant strategy to counteract obesity and related comorbidities.

## Methods

### Animals

Adult male C57 BL/6 and *ob/ob* mice were purchased from the National Resource Center of Model Mice (Nanjing, China), and housed under a 12-h light/12-h dark cycle in a temperature-controlled room (22–24 °C). Oxt-Cre mice were obtained from the Jackson Laboratory (Bar Harbor, ME)^32^. The animals had ad libitum access to tap water and diet, except where noted. All of the experimental procedures were approved by the Institutional Animal Care and Use Committee of the Huazhong University of Science and Technology. Rodent chow (9.4% kcal from fat) and high-fat (60% kcal from fat) diets were purchased from the HFK Bioscience (Beijing, China) and Medicience (Yangzhou, China), respectively. For diet-induced obesity, mice were fed the HFD for 12 weeks starting at 6 weeks of age unless otherwise noted.

### Antibodies and chemicals

The detailed information of antibody is provided in Supplementary Table 1. Rabbit anti-A_1_R antibody was purchased from Alomone labs (Jerusalem, Israel). Goat anti-A_1_R, anti-c-Fos, anti-CRH, anti-Neurophysin I and anti-Neurophysin II, rabbit anti-c-Fos, anti-TRH, anti-A_2A_R, anti-A_3_R and anti-p-Creb1 (Ser 133), and mouse anti-β-Actin and anti-HA antibodies were obtained from Santa Cruz Biotechnology (Santa Cruz, CA). Mouse anti-Hu C/D and anti-Myc antibodies were purchased from Thermo Fisher (Waltham, MA) and Proteintech (Wuhan, China), respectively. Rabbit anti-Oxt antibody was obtained from Immunostar (Hudson, WI). Rabbit anti-A_2B_R and anti-AVP antibodies were purchased from Bioss (Woburn, MA). Alexa Fluor 488/555 goat anti-rabbit, Alexa Fluor 488/555 goat anti-mouse, Alexa Fluor 488/555 donkey anti-rabbit and Alexa Fluor 488/555/633 donkey anti-goat secondary antibodies were also obtained from Thermo Fisher. Adenosine was obtained from Aladdin (Shanghai, China). Caffeine, CPA and Avertin was purchased from Amresco (Solon, OH), Tocris (Bristol, UK) and Sigma (St Louis, MO), respectively. Oxt was purchased from Sangon Biotech (Shanghai, China). OTR inhibitor L-368,899 was obtained from Santa Cruz.

### Plasmid construction and lentivirus production

To generate a neuron-specific lentiviral vector, we replaced the cytomegalovirus promoter in the Lentilox 3.7 vector by the human *Synapsin* promoter^25^. This plasmid expressed EGFP in neurons and was designated as Ctrl-Lenti. We amplified the full-length mouse A_1_R cDNA with gene-specific primers (Supplementary Table 2) and ligated the fragment to Ctrl-Lenti at the NheI site. This A_1_R-expressing plasmid was designated as A_1_R-Lenti. To generate pcDNA3 HA-A_1_R, HA-tagged mouse A_1_R cDNA was cloned into pcDNA3 between the HindIII and EcoRI restriction sites. shA_1_R-Lenti plasmid was constructed by inserting shRNA-expressing cassette (Supplementary Table 2) to Lentilox 3.7 between the HpaI and XhoI sites^63^. shA_1_R-pSico lentiviral plasmid was constructed by using the same expression cassette as previous described^31^. The corresponding empty vectors were used as controls. Successful constructions of the plasmids were verified by DNA sequencing. Lentivirus was produced in HEK293T cells by transfecting the cells with lentiviral and packaging plasmids, and concentrated by using ultracentrifugation^14^.

### Surgery

Third ventricle cannulation: stereotacxic surgery was performed as previously described^14^,^18^. Briefly, mouse was anaesthetized with sodium pentobarbital (75 mg kg^−1^) or Avertin (300 mg kg^−1^) and placed on an ultra-precise stereotaxic instrument (David Kopf, Tujunga, CA). Then a 28 G guide cannula was implanted targeting the ventral third ventricle (coordinates: A/P -2.0 mm posterior to bregma, D/V −5.0 mm). For double cannula directed to PVN, the coordinates were A/P −0.85 mm posterior to bregma, D/V −4.2 mm. Mice were allowed to recover for 2 weeks and successful implantation was shown by Angiotensin II evoked water-drinking behaviour. For lentivirus injection, mice were anaesthetized and placed on the stereotaxic instrument. With the help of a guide cannula, the viral solution was injected bilaterally to PVN, ARC or DMH at the coordinates −0.85, −1.80 or −1.90 mm posterior to, 0.2, 0.2 or 0.3 mm lateral to, and −4.8, −5.8 or −5.0 mm below bregma.

### I.C.V. administration of reagents

Caffeine: HFD-fed mice were cannulated and allowed to recover from surgeries, and then caffeine was administered at the dose of 10 μg per mouse immediately before the light was turned off. Food intake and body weight were measured on a daily basis (Supplementary Fig. 9k). The mean daily consumption of food was presented. To initially characterize the dose-response effect, we administered 1, 5, 10 or 15 μg of caffeine to mouse brains and measured the consumed foods in 24 h.

Adenosine and CPA: chow-fed mice were cannulated, allowed to recover and then injected the indicated doses of adenosine, CPA (50 ng per mouse) or control immediately before the dark cycle. Four and 24 h food intakes were measured.

Caffeine or Oxt administered to A_1_R-Lenti or Ctrl-Lenti virus-injected mice: virus-injected and 3rd ventricle-cannulated mice were given caffeine (10 μg per mouse) or Oxt (1 μg per mouse)^27^, and aCSF as control. Twenty-four hours food intake and body weight change were measured.

OTR antagonist, AVP or TRH antibody and caffeine administered to DIO mice: Third ventricle-cannulated DIO mice were i.c.v. administered OTR antagonist L-368,899 (2 μg per mouse), AVP antibody (0.5 μg per mouse), TRH antibody (0.5 μg per mouse)^64^ or aCSF/non-immune IgG as control. An hour later and immediately before the light was turned off, caffeine (10 μg per mouse) or aCSF was injected. Twenty-four hours food intake and body weight change were measured and presented.

### Intra-PVN injection of caffeine

DIO mice were implanted double cannula directed to both sides of PVN. After the mice were recovered form surgeries, caffeine was administered at the doses up to 1 μg per mouse immediately before the dark cycle. Twenty-four hours food intake and body weight change were measured.

### Oral gavage of caffeine

Caffeine (60 mg kg^−1^) and saline were delivered to the DIO mice by using a gavage needle immediately before the dark cycle. Daily food intake and body weight were measured (Supplementary Fig. 10e). The mean daily food intake was presented. To characterize the dose-response effect, we administered caffeine at the indicated doses, and measured the consumed HFD in 24 h.

### Indirect calorimetry and locomotor activity

O_2_ consumption, CO_2_ production and energy expenditure were measured by using the OxyletPro indirect calorimetry system (Panlab, Barcelona, Spain). We defined the value immediately before the delivery of caffeine or control as basal value. The parameters were normalized by lean body mass (lbm), which was measured by using a Minispec LF50 body composition analyzer (Bruker, Rheinstetten, Germany). For wheel-running locomotor activity, mice were housed in cages with steel wheels (diameter=11.5 cm), and were accustomed to the cages and running wheels for 3 days. Reagents were administered at 11:00 AM and the revolution number was recorded by a digital counter connected to a magnet sensor.

### Glucose tolerance test and plasma TG analysis

Glucose tolerance test (GTT) was performed as previously described^14^. Briefly, mice were fasted overnight and intraperitoneally administered glucose (2 g kg^−1^). Blood glucose levels were measured by using a glucometer at the indicated time points (Omron, Beijing, China). Plasma TG analysis: fasting plasma TG was determined with reagent from Jiancheng Bioengineering Institute (Nanjing, China) according to the manufacture's instruction.

### Thermography

Infrared images were taken from lentivirus-injected mice, or 4 h post i.c.v. administration of aCSF or caffeine at 22 °C with an infrared camera (Flir, Boston, MA). Data were analysed with Tool+ software (Flir, Boston, MA). Mean of the highest 10% values in the interscapular area was presented as previously described^65^.

### Behavioural analyses

Open field test: mice were administered aCSF or caffeine (10 μg per mouse) 2 h before the test. The animals were then placed in an opaque, square open field (40 cm L × 40 cm W × 40 cm H). Mice were allowed to freely explore in the field for 5 min and monitored with the ImageOF software (https://cbsn.neuroinf.jp/modules/xoonips/detail.php?id=ImageOF). The open field was divided into a peripheral region and a 13.3 cm × 13.3 cm central region. Time spent in the central versus peripheral region of the field during the 5 min period was presented.

Elevated plus maze test: the plus maze had two walled arms (the closed arms, 35 cm L × 6 cm W × 22 cm H) and two open arms (35 cm L × 6 cm W). The maze was elevated 74 cm from the floor. Mice were placed on the center section and allowed to explore the maze freely and monitored with ImageEP software^66^. Time spent in the open versus closed arms during the 5 min period was presented.

Light/dark box text: the apparatus was comprised of a dual compartment box (20 cm L × 20 cm W × 40 cm H) with free access between them. The dark box was made of black Plexiglass and the light one was exposed to room light. The exploratory activity was monitored for 5 min by using the ImageLD software^67^. Time spent in the light versus dark box was presented.

Sleep/wakefulness test: caffeine (10 μg per mouse) or aCSF was i.c.v. administered immediately before the dark cycle. Mice were monitored in home cage with a camera during the first 4 h of light cycle. Sleep durations were calculated when animals were immobilized for 40 s or longer by manually inspecting the videos^68^.

Time spent in food intake or non-food intake-related locomotor activity. Caffeine (10 μg per mouse) or aCSF was i.c.v. injected immediately before the dark phase. Mice were monitored in home cage with an infrared camera (Flir, Boston, MA) during the first 4 h of the dark cycle. Time spent in food intake or other locomotor activities were calculated by manually inspect the videos.

### Histology

Mice were administered control or caffeine (i.c.v., 10 μg per mouse; oral gavage, 60 mg kg^−1^). Two hours later, mice were anaesthetized by sodium pentobarbital and fixed with 4% paraformaldehyde via transcardial perfusion. Brain tissues were cryoprotected by 20% and 30% sucrose solutions, and sectioned on a cryostat. Tissue sections were blocked with 5% serum/0.3% Triton X-100/PBS, incubated with primary antibodies at 4 °C overnight, and fluorophore-labelled secondary antibodies at room temperature for 1 h. For double immunostaining of TRH or CRH and A_1_R, mice were i.c.v. administered colchicine (40 μg per mouse) 2 days before the perfusion. Immunofluorescence staining of cell: HEK293T cells were plated on cover glasses and transfected with pcDNA3 HA-A_1_R or control plasmid. Two days later, cells were washed with 1 × PBS and fixed with 4% PFA. Immunostaining of A_1_R and HA antibodies were then performed. Image sets encompassing only c-Fos and DAPI were collected with a conventional AE31 fluorescent microscope (Motic, Xiamen, China). Other fluorescent images were acquired with a FluoView FV1200 confocal microscope (Olympus, Tokyo, Japan). All of the images were processed with ImageJ software. The number of c-Fos^+^, A_1_R^+^, NP-Oxt^+^ and double positive cells were manually counted in one side of the nucleus of one representative brain section per mouse.

Haematoxylin and eosin staining: epididymal white adipose tissue tissues were removed and fixed in Bouin’s solution, and embedded in paraffin. Tissues were sectioned and stained with haematoxylin and eosin solutions sequentially. Images were collected on the AE31 microscope. We measured the size of adipocyte by using the ImageJ.

### Analysis of adenosine level

Twenty-two weeks HFD or chow-fed, *ob*/*ob* and wild-type C57 BL/6 mice were implanted cannulas (0.48 mm OD × 0.34 mm ID) directed to the third ventricle, and allowed to recover from surgeries. Freely released fluid was then collected as CSF. Plasma and Hypothalamic tissues were collected from 24 weeks HFD-fed mice and matched controls. Hypothalamic tissues were lysed with tissue lysis buffer. Protein concentrations were then determined. Plasma, CSF and hypothalamic tissue samples were measured by using an adenosine assay kit obtained from Biovision (Milpitas, CA). Hypothalamic adenosine content was normalized against the amount of protein.

### qRT-PCR

Tissue RNA was extracted by using the TRIzol reagent (Thermo Fisher, Waltham, MA). cDNA was generated with MMLV reverse transcriptase (Promega, Madison, WI). After the combination of cDNA, gene-specific primers (Supplementary Table 2) and PCR master mix (Thermo Fisher, Waltham, MA), the assay was performed on an ABI 7900HT real-time PCR system (Thermo Fisher, Waltham, MA). We used the 2^−ΔCt^ method to analyse the relative expression level of genes, where ΔCt is the difference between the Ct value of a given gene and that of Gapdh control. 

### Western blot and co-immunoprecipitation

Western blot: proteins were extracted from hypothalamic tissues, separated by SDS-PAGE, and transferred to PVDF membranes. The membranes were then blocked by 5% non-fat milk and incubated with rabbit anti-A_1_R (1:1,000), anti-A_2A_R (1:1,000), anti-A_2B_R (1:1,000) or anti-A_3_R (1:500) antibody, or mouse anti-β-Actin antibody (1:2,000). After incubation with horseradish peroxidase labelled secondary antibody (1:4,000), the membranes were exposed to the Supersignal West Femto Maximum Sensitivity Substrate (Thermo Fisher, Waltham, MA). Chemiluminescence was recorded with the GeneGnome system (Syngene, Cambridge, UK). Co-immunoprecipitation: HEK293T cells were transfected with pcDNA3 HA-A_1_R and Myc-OTR (Origene, Rockville, MD) expressing plasmids. Cell lysates were incubated with 2 μg of non-immune IgG, anti-HA or anti-Myc antibody. Immunoprecipitates were prepared by incubation with protein A/G-agarose (Santa Cruz Technology, Santa Cruz, CA), and subjected to western blot with either anti-Myc or anti-HA antibody. Uncropped blot images with molecular weight reference are shown in Supplementary Fig. 16 through 18.

### Single-cell RT-PCR

For single-cell RT-PCR, mice were fed the chow or high-fat diet for 24 weeks. The animals were euthanized with sodium pentobarbital overdose and brains were removed and sectioned to slices at 300 μm thickness. The brain slices encompassing PVN were then treated with papain (Worthington Biochemical, Lakewood, NJ) and cells in the PVN region were harvested by using a capillary pipette filled with 1 × buffer for reverse transcription. The cells were then expelled to a 0.2 ml tube containing dNTPs, Oligo(dT)_15_ primer and MMLV reverse transcriptase. The mixtures were incubated at 42 °C to generate cDNA. We performed a two-stage RT-PCR to detect A_1_R, Oxt, AVP and TRH. In each stage there were 35 cycles for amplification. The internal control Gapdh was amplified in one stage. PCR products were visualized with ethidium bromide on a 2% agarose gel. Uncropped gel images with molecular weight reference are presented in Supplementary Fig. 19. All primers used are listed in Supplementary Table 2.

### Oxytocin release assay

Caffeine: to determine the effect of caffeine on Oxt release, PVN slices from HFD-fed mice were dissected and balanced in Locke solution supplied with 95% O_2_ and 5% CO_2_ at 37 °C. The solution was changed every 5 min for 10 times and the 10th sample was collected to measure the basal release rate. The tissues were then incubated in the same solution containing caffeine (2 mmol l^−1^)^69^ for 5 min and this solution was measured to determine caffeine-evoked Oxt release.

Effect of overexpression of A_1_R in PVN on Oxt release: the detailed procedure has been described previously^18^. Briefly, A_1_R-Lenti or Ctrl-Lenti virus was injected into the PVN of chow-fed mice. The PVN slices were then dissected and incubated in the Locke solution for 10 times. The tissues were incubated in High K^+^ Locke solution containing 70 mmol l^−1^ KCl for 5 min and this solution was measured to determine KCl stimulated Oxt release. An EIA kit (Enzo, Farmingdale, NY) was used to determine the Oxt concentration in the solution.

### Statistical analysis

Data are expressed as mean±s.e.m. The two-tailed Student's *t*-test was used to compare two groups. One-way analysis of variance followed by the Bonferroni's or Newman–Keuls *post hoc* test was used for comparisons of more than 2 groups. Two-way analysis of variance followed by the Bonferroni's *post hoc* test was used for multiple comparisons. Linear regression was employed to analyse the relationship between adenosine levels and body weights. Pearson’s correlation coefficient and the best-fit line were calculated. *P*<0.05 was considered statistically significant.

### Data availability

All data generated or analysed during this study are included in this published article (and its Supplementary Information file), or available from the corresponding author upon reasonable request.

## Author cntributions

L.W. and J.M. designed and performed the experiments as well as analysed the data. Q.S., Y.Z., S.P., Z.C. and Y.H. performed the experiments and analysed the data. L.-Q.Z. and Y.L. provided technical support. G.Z. conceived the study, designed the experiments, analysed the data and wrote the manuscript. All authors commented on the manuscript.

## Additional information

**How to cite this article:** Wu, L. *et al*. Caffeine inhibits hypothalamic A_1_R to excite oxytocin neuron and ameliorate dietary obesity in mice. *Nat. Commun.* **8,** 15904 doi: 10.1038/ncomms15904 (2017).

**Publisher’s note:** Springer Nature remains neutral with regard to jurisdictional claims in published maps and institutional affiliations.

## Supplementary Material

Supplementary Information
