# Supplementary material for: Caffeine inhibits hypothalamic A1R to excite oxytocin neuron and ameliorate dietary obesity in mice
Source: Nat Commun. 2017 Jun 27;8:15904. doi: 10.1038/ncomms15904 (PMC5490268; doi:10.1038/ncomms15904)
Supplement: Supplementary Information [file ncomms15904-s1.pdf]

File name: Supplementary Information
Description: Supplementary Figures and Supplementary Tables

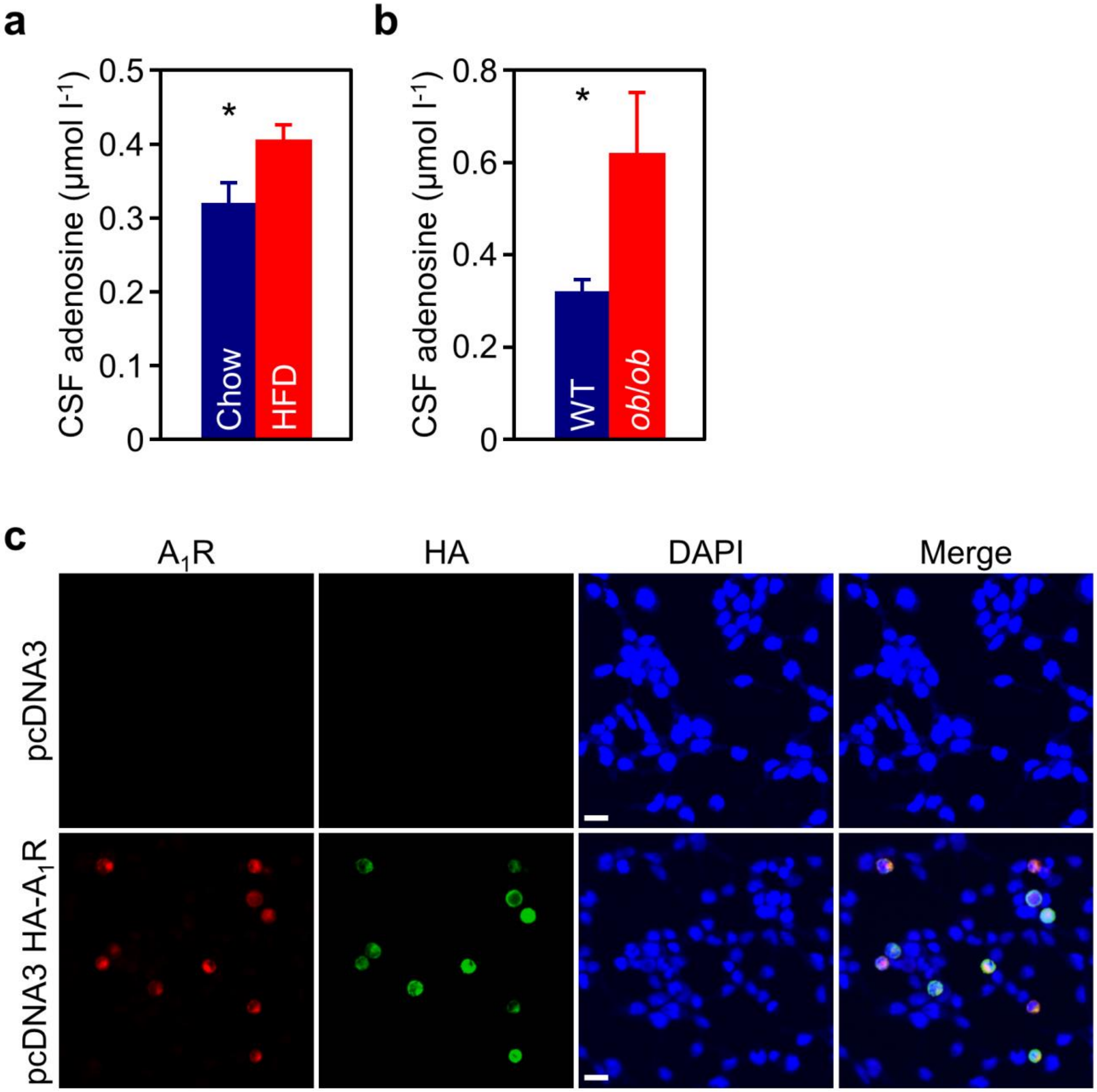

Supplementary Fig. 1. Elevated levels of CSF adenosine in short term HFD-fed and *ob*/*ob* mice, and the validation of rabbit anti-A_1R antibody. (**a**) Adenosine levels in the CSF of chow- or 2 weeks HFD-fed mice. n=7. (**b**) Adenosine levels in the CSF of 2 months old wild type and *ob/ob* mice. n=7. (**c**) Validating the specificity of the rabbit anti-A_1R antibody**.** HEK293T cells were transfected with pcDNA3 as control, or pcDNA3 HA-A_1R plasmid, which expressed HA-tagged mouse A_1R. Double immunofluorescence staining of A_1R (red) and HA (green) were performed. Cell nuclei were counterstained with DAPI (blue). Scale bar, 20 μm. * p<0.05, two-tailed Student's t test.

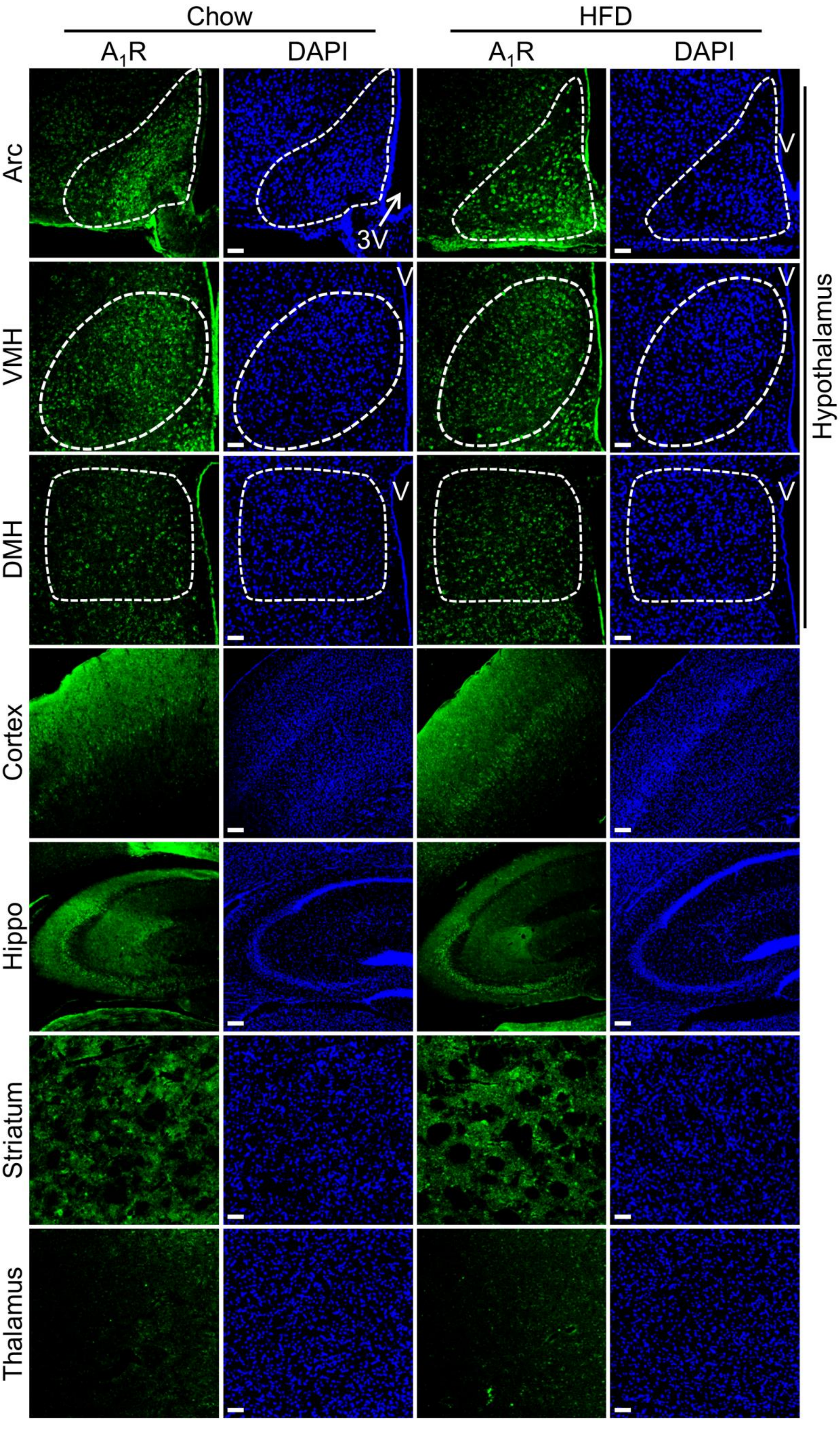

Chow
HFD
A1R
DAPI
A1R
DAPI
Arc
VMH
DMH
Cortex
Hippo
Striatum
Thalamus
Hypothalamus
3V
V

Supplementary Fig. 2. Expression of A_1R in the brain of mouse fed a regular chow or HFD. Adult male C57 BL/6 mice were fed chow or HFD for 12 weeks. The mice were anesthetized with sodium pentobarbital and transcardially perfused by using 4% PFA. Mouse brains were cryoprotected by sucrose solutions and cryo-sectioned. Rabbit anti-A_1R (green) antibody and Alexa Fluor 488 goat anti-rabbit secondary antibodies were sequentially applied. Tissue sections were mounted in aqueous medium with DAPI (blue). Immunofluorescence was visualized under a confocal microscope. **Arc**, Arcuate nucleus of hypothalamus; **VMH**, Ventromedial nucleus of hypothalamus; **DMH**, Dorsomedial nucleus of hypothalamus; **Hippo**, Hippocampus. **V** and **3V**, third ventricle. Scale bar, 50 μm.

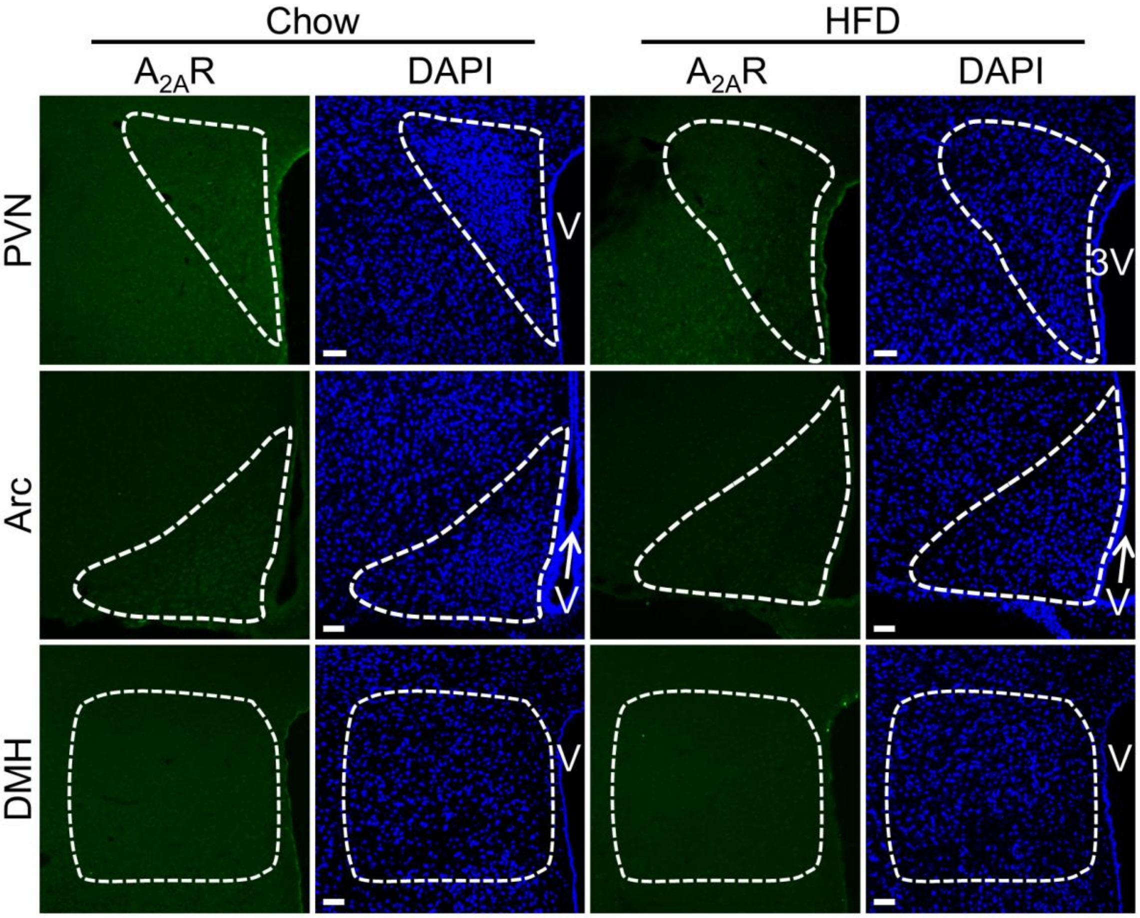

Supplementary Fig. 3. Expression of $A_{2A}R$ in the hypothalamus of mouse fed a regular chow or HFD. Immunofluorescence staining of $A_{2A}R$ (green) in the paraventricular nucleus of hypothalamus (**PVN**), Arc and DMH of mice fed a regular chow or HFD for 12 weeks. Cell nuclei were counterstained with DAPI (blue). **V** and **3V**, third ventricle. Scale bar, 50 μm.

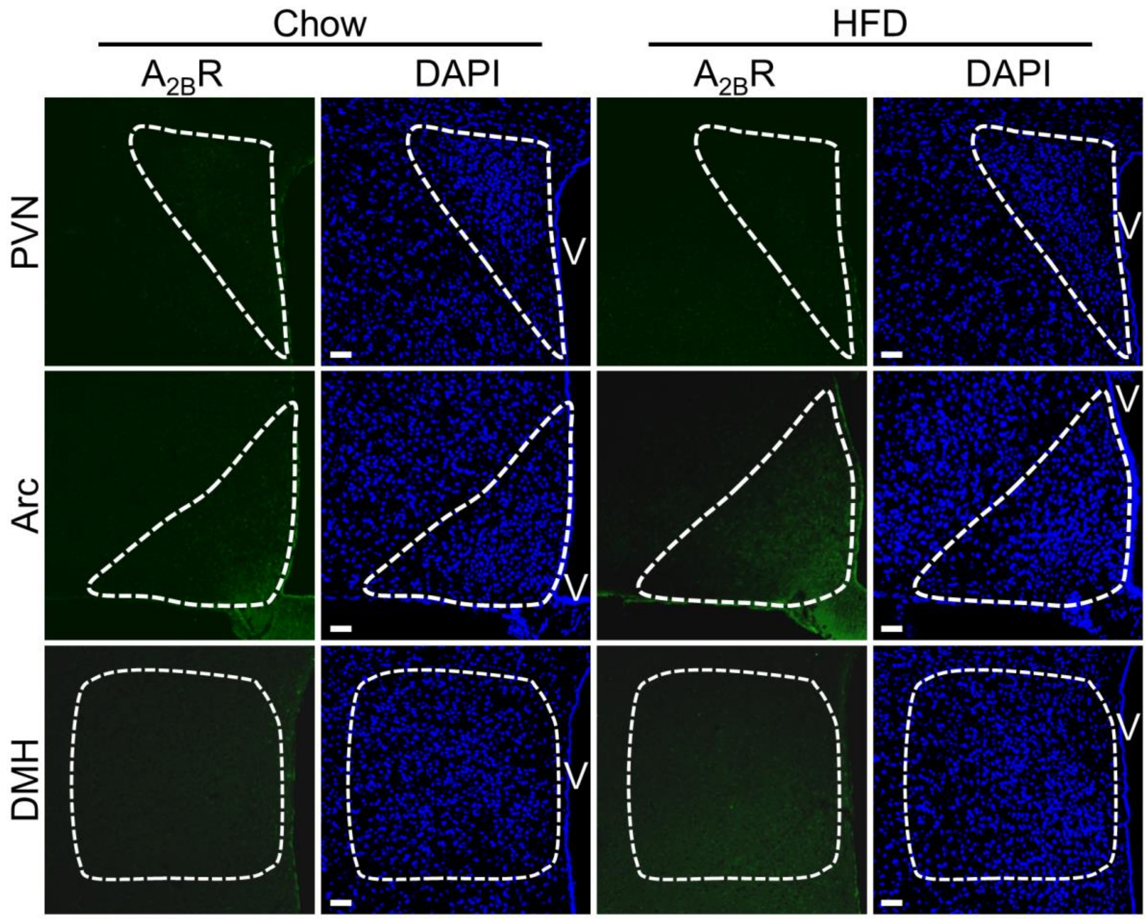

Supplementary Fig. 4. Expression of $A_{2B}R$ in the hypothalamus of mouse fed a regular chow or HFD. Immunofluorescence staining of $A_{2B}R$ (green) in the PVN, Arc and DMH of mice fed chow or HFD for 12 weeks. Cell nuclei were counterstained with DAPI (blue). **V**, third ventricle. Scale bar, 50 μm.

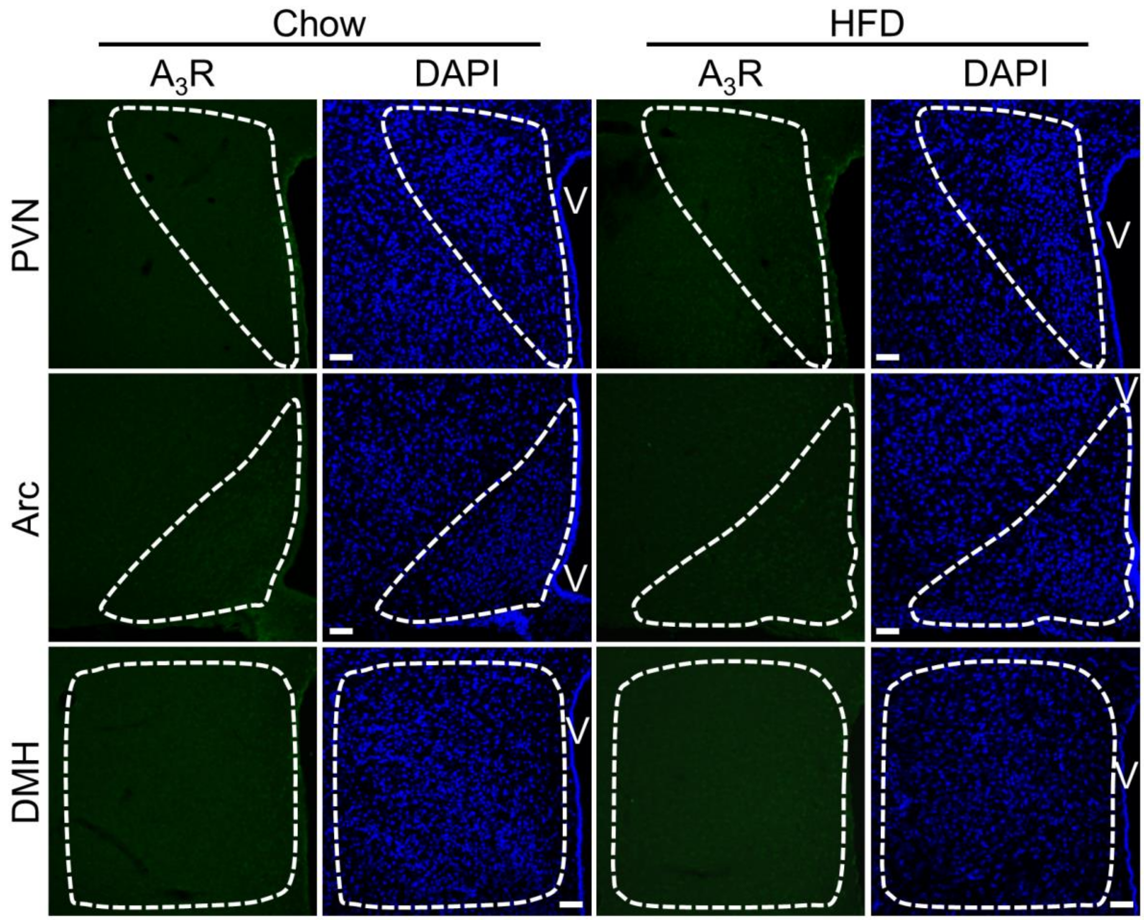

Supplementary Fig. 5. Expression of A_3R in the hypothalamus of mouse fed a regular chow or HFD. Immunofluorescence staining of A_3R (green) in the PVN, Arc and DMH of mice fed a regular chow or HFD for 12 weeks. Cell nuclei were counterstained with DAPI (blue). **V**, third ventricle. Scale bar, 50 μm.

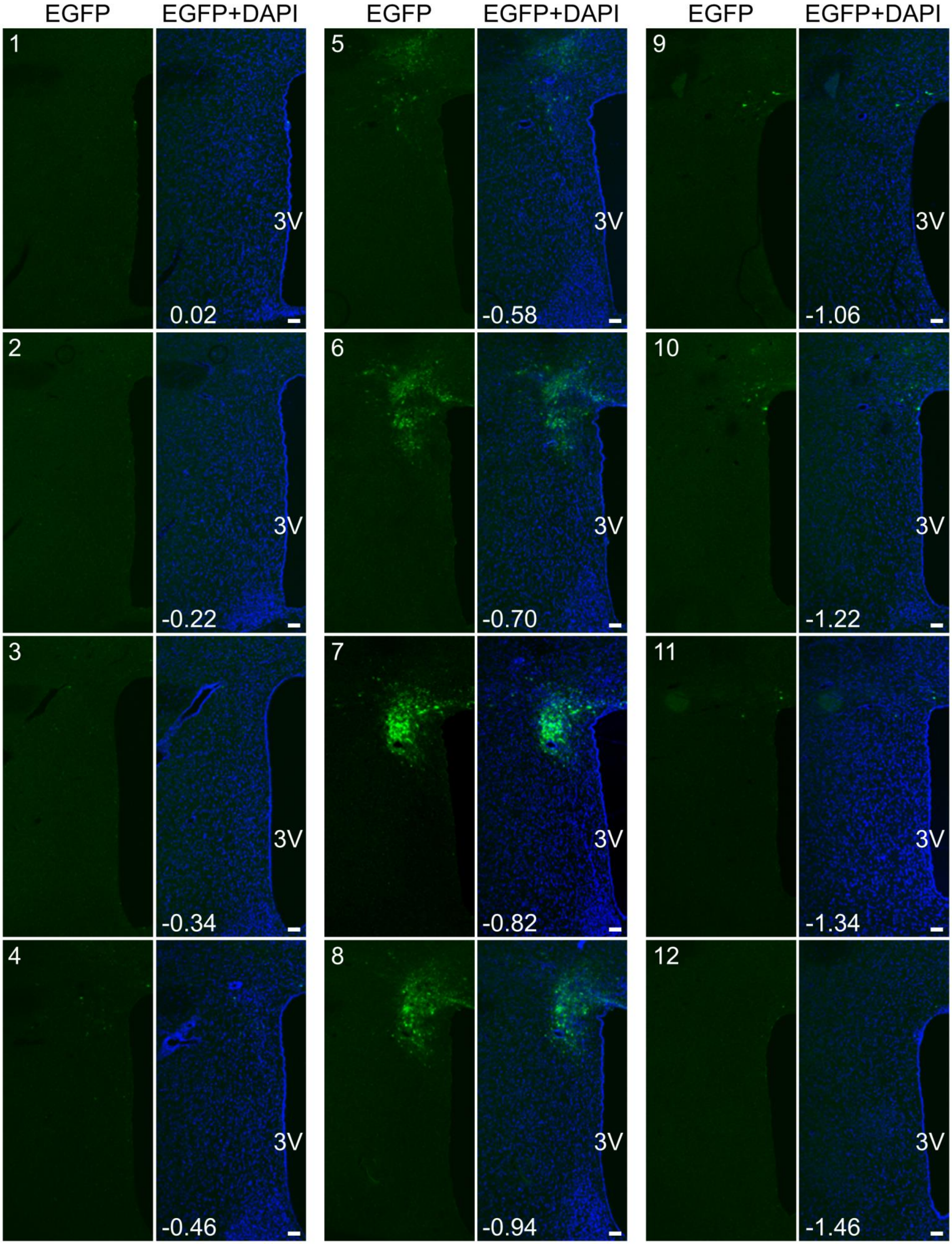
EGFP
EGFP+DAPI
EGFP
EGFP+DAPI
EGFP
EGFP+DAPI
1
0.02
2
-0.22
3
-0.34
4
-0.46
5
-0.58
6
-0.70
7
-0.82
8
-0.94
9
-1.06
10
-1.22
11
-1.34
12
-1.46
3V

Supplementary Fig. 6. Expression pattern of EGFP in mouse brain after the injection of Ctrl-Lenti virus. Adult male C57 BL/6 mouse was injected Ctrl-Lenti virus to the PVN. The Ctrl-Lenti virus led to the expression of EGFP driven by the human *Synapsin* promoter. The animal was transcardially perfused, and brain was dissected and sectioned (30 μm) coronally by using a cryostat. Sections were shown in the rostral-to-caudal direction (#1 to #12). The number at the bottom of each merged image indicates the position relative to Bregma (Franklin and Paxinos, The Mouse Brain in Stereotaxic Coordinates, 3rd ed., 2007). Cell nuclei were counterstained with DAPI (blue). **3V**, third ventricle. Scale bar, 50 μm.

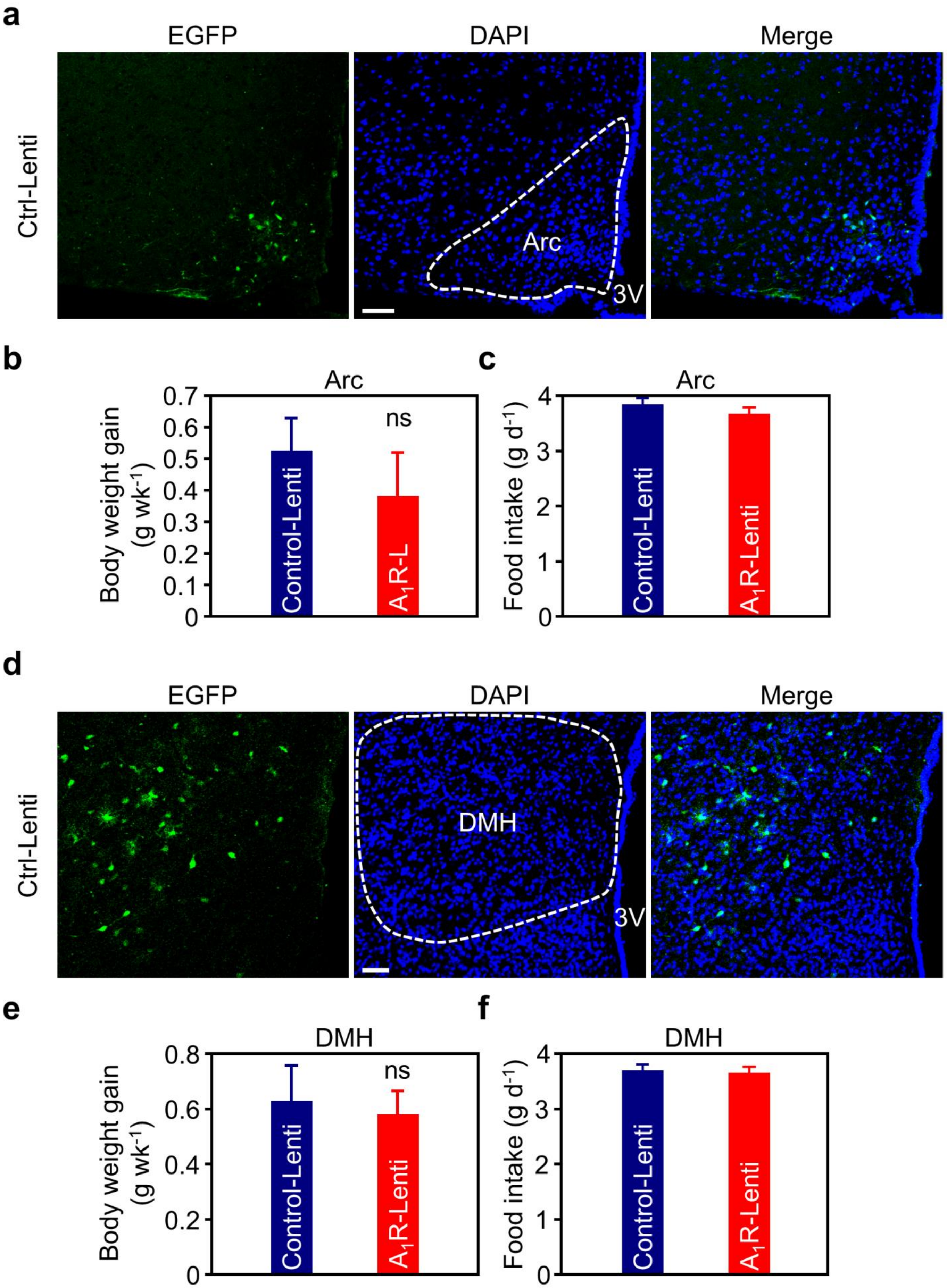
a
EGFP
DAPI
Merge
Ctrl-Lenti
Arc
3V
b
Arc
Body weight gain (g wk^{-1})
0.7
0.6
0.5
0.4
0.3
0.2
0.1
0
ns
Control-Lenti
A_1R-L
c
Arc
Food intake (g d^{-1})
4
3
2
1
0
Control-Lenti
A_1R-Lenti
d
EGFP
DAPI
Merge
Ctrl-Lenti
DMH
3V
e
DMH
Body weight gain (g wk^{-1})
0.8
0.6
0.4
0.2
0
ns
Control-Lenti
A_1R-Lenti
f
DMH
Food intake (g d^{-1})
4
3
2
1
0
Control-Lenti
A_1R-Lenti

Supplementary Fig. 7. Effects of overexpression of A_1R in the neurons of Arc or DMH on energy balance. A_1R-Lenti (**A_1R-L**) or Ctrl-Lenti virus was injected into the Arc or DMH nuclei of chow-fed mice. (**a**) Expression of EGFP (green) after the injection of Ctrl-Lenti to Arc is shown. (**b,c**) Body weights and food intakes of mice injected lentivirus to Arc were monitored for 3 weeks, and the mean weekly body weight gain (**b**) and daily food intake (**c**) are presented. (**d**) Expression of EGFP after the injection of Ctrl-Lenti to DMH is shown. (**e**,**f**) Body weights and food intakes of mice injected lentivirus to DMH were monitored for 3 weeks, and the mean weekly body weight gain (**e**) and daily food intake (**f**) are presented. Cell nuclei were counterstained with DAPI (blue). **3V**, third ventricle. Scale bar, 50 μm. For ARC, n= 7 (Ctrl-Lenti), 6 (A_1R-Lenti). DMH: n= 7 (Ctrl-Lenti), 8 (A_1R-Lenti). **ns**, not significant, two-tailed Student's t-test (**b**,**e**).

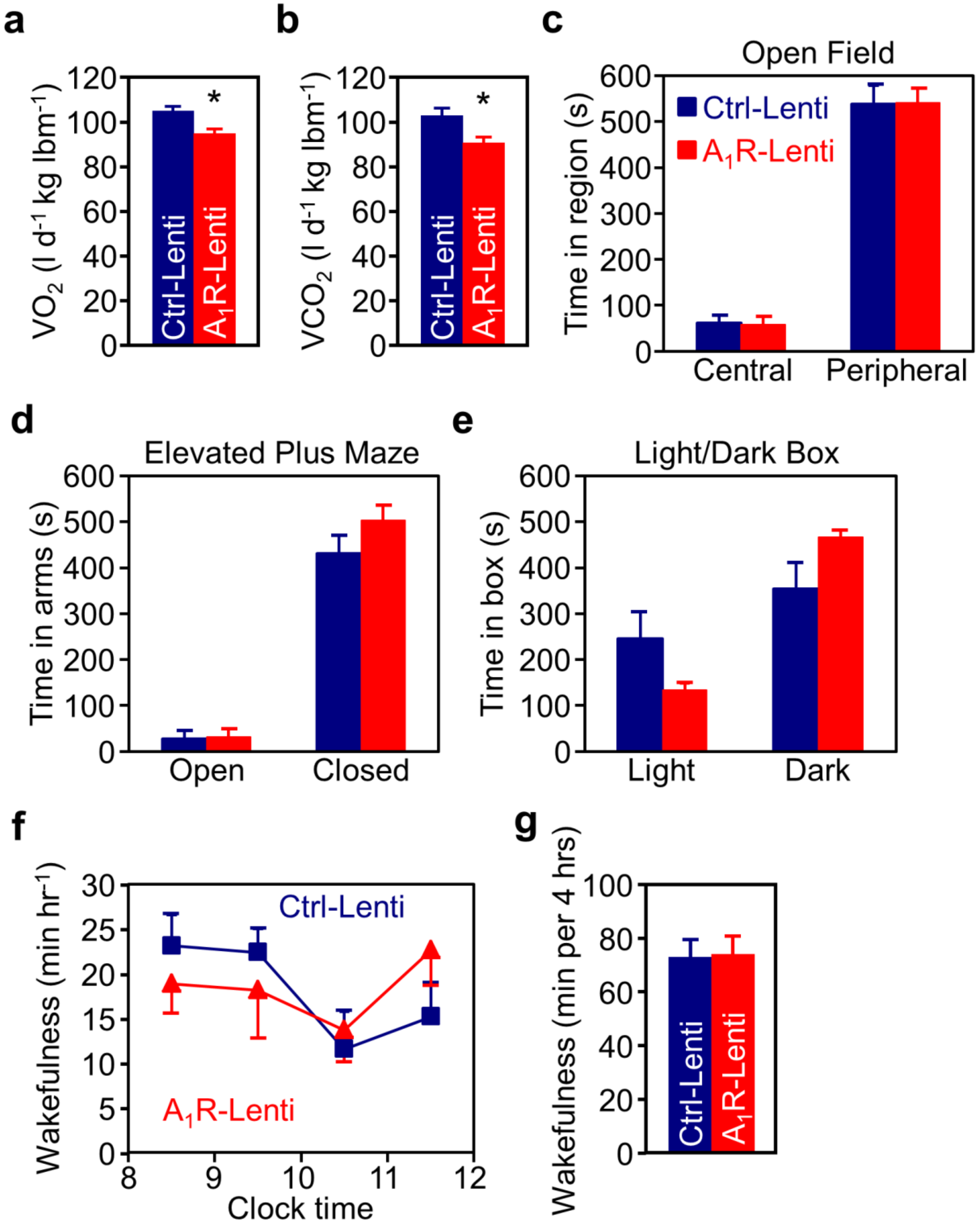

Supplementary Fig. 8. Effects of overexpression of A_1R in PVN neurons on energy expenditure and mouse behaviors. (**a**-**b**) O_2 consumption (**a**) and CO_2 production (**b**) of Ctrl-Lenti or A_1R-Lenti injected mice. **lbm**, lean body mass. n=6. (**c**-**e**) A_1R-Lenti and Ctrl-Lenti viruses were injected into the PVN of chow-fed mice , respectively. Open field (**c**), elevated plus maze (**d**) and light/dark box (**e**) tests were then performed. No significant difference was observed between the two groups. n=7-10. (**f**-**g**) Wakefulness time of Ctrl-Lenti or A_1R-Lenti injected mice were measured during the first 4 hours of the light cycle (8 am-12 pm). n=8. *$p<0.05$, two-tailed Student's t-test (**a**,**b**).

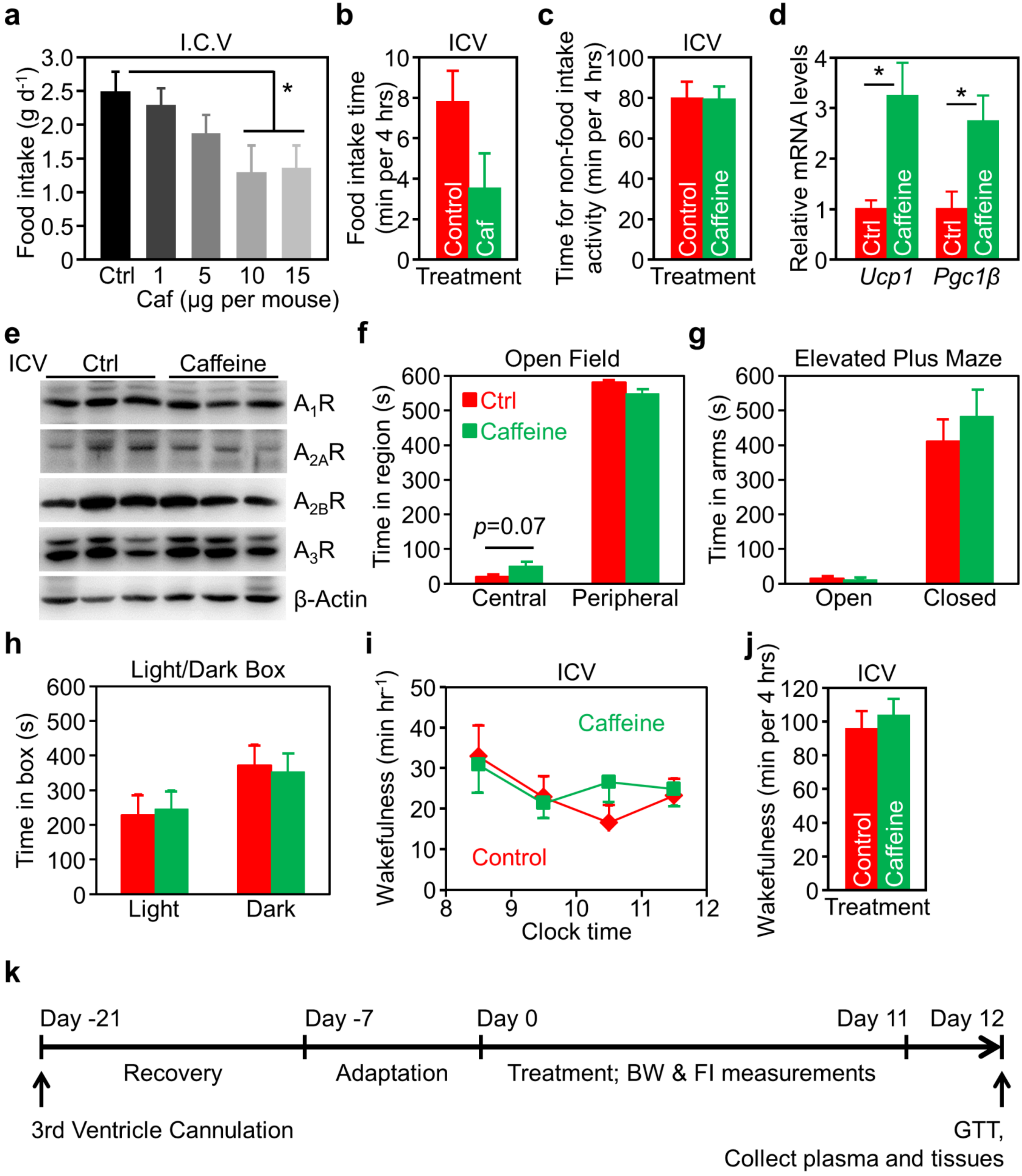
a
I.C.V
Food intake (g d-1)
*
Ctrl 1 5 10 15
Caf (μg per mouse)
b
ICV
Food intake time (min per 4 hrs)
Control
Caf
Treatment
c
ICV
Time for non-food intake activity (min per 4 hrs)
Control
Caffeine
Treatment
d
Relative mRNA levels
Ctrl
Caffeine
Ucp1 Pgc1β
e
ICV
Ctrl
Caffeine
A1R
A2AR
A2BR
A3R
β-Actin
f
Open Field
Time in region (s)
Ctrl
Caffeine
p=0.07
Central Peripheral
g
Elevated Plus Maze
Time in arms (s)
Open Closed
h
Light/Dark Box
Time in box (s)
Light Dark
i
ICV
Wakefulness (min hr-1)
Caffeine
Control
Clock time
j
ICV
Wakefulness (min per 4 hrs)
Control
Caffeine
Treatment
k
Day -21
Day -7
Day 0
Day 11
Day 12
Recovery
Adaptation
Treatment; BW & FI measurements
3rd Ventricle Cannulation
GTT,
Collect plasma and tissues

Supplementary Fig. 9. Effects of i.c.v. administered caffeine on food intake and mouse behaviors. (**a**) Dose-dependent effects of i.c.v. administered caffeine on HFD intake. n=7-8. (**b**-**c**) Time spent on food intake (**b**) or non-food intake-related locomotor activity (**c**) was measured during the first 4 hours of the dark cycle. n=6 (Control), 7 (Caf). (**d**) Relative mRNA levels of Ucp1 and Pgc1β of mice i.c.v. administered control or 10 μg of caffeine. n=7. (**e**) Western blot analysis of adenosine receptor expression in the hypothalami of mice i.c.v. administered control or caffeine for 11 days. β-Actin was used as loading control. (**f**-**h**) Open field (**f**), elevated plus maze (**g**) and light/dark box tests (**h**) were performed 2 hours after the mice were i.c.v. injected control or caffeine. n=8. (**i**-**j**) Wakefulness time of control or caffeine injected mice were measured during the first 4 hours of the light cycle. Caffeine and aCSF were injected immediately before the previous dark cycle. n=8. (**k**) Schematic diagram of the experimental procedures of brain caffeine treatment. Mice were given aCSF by using the i.c.v. route during the adaptation period. **BW**, body weight. **FI**, food intake. *$p<0.05$, one way ANOVA (**a**) or two-tailed Student's t-test (**d**).

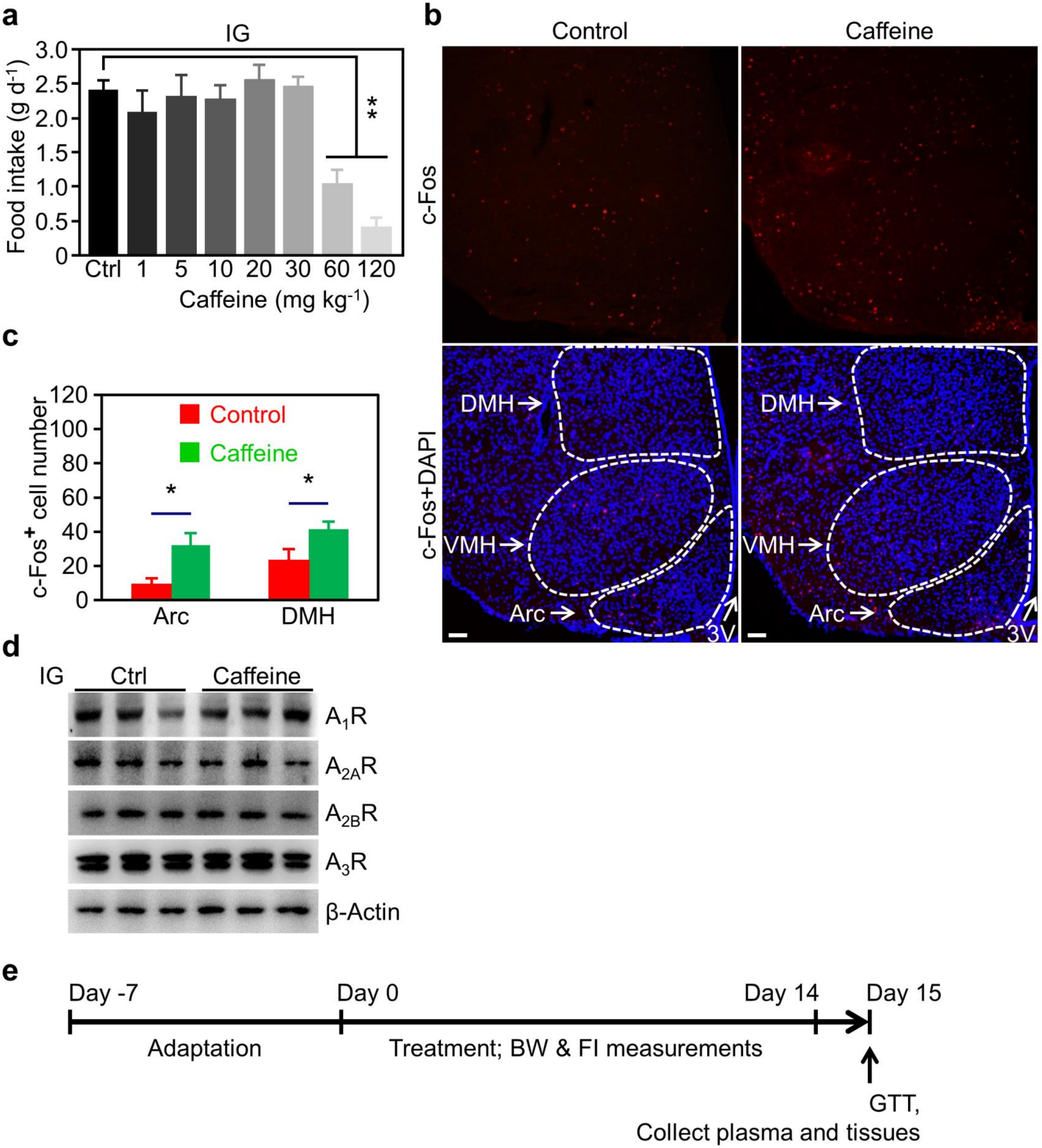
a
IG
Food intake (g d-1)
3.0
2.5
2.0
1.5
1.0
0.5
0
**
Ctrl
1
5
10
20
30
60
120
Caffeine (mg kg-1)
b
Control
Caffeine
c-Fos
c-Fos+DAPI
DMH
VMH
Arc
3V
c
c-Fos+ cell number
120
100
80
60
40
20
0
Control
Caffeine
*
*
Arc
DMH
d
IG
Ctrl
Caffeine
A1R
A2AR
A2BR
A3R
β-Actin
e
Day -7
Day 0
Day 14
Day 15
Adaptation
Treatment; BW & FI measurements
GTT,
Collect plasma and tissues

Supplementary Fig. 10. (**a**) Dose-response effect of intragastrically (**IG**) administered caffeine on HFD intake. n=7-9. (**b**) Immunostaining of c-Fos (red) in the Arc, VMH and DMH nuclei of mice injected control or caffeine (60 mg kg^{-1}) by using oral gavage. Cell nuclei were counterstained with DAPI (blue). **3V**, third ventricle. Scale bar, 50 µm. (**c**) Numbers of c-Fos^{+} cells in the Arc and DMH nuclei. n=7 (Control), 6 (Caffeine). (**d**) The protein levels of the adenosine receptors in the hypothalami of mice peripherally administered caffeine or saline for 2 weeks. β-Actin was used as loading control. (**e**) Schematic diagram of the experimental procedures of peripheral caffeine treatment. Mice were given saline by using oral gavage during the adaptation period. **BW**, body weight. **FI**, food intake. **p<0.01, one-way ANOVA with Bonferroni's post-hoc test (**a**). *p<0.05, two-tailed Student's t-test (**c**).

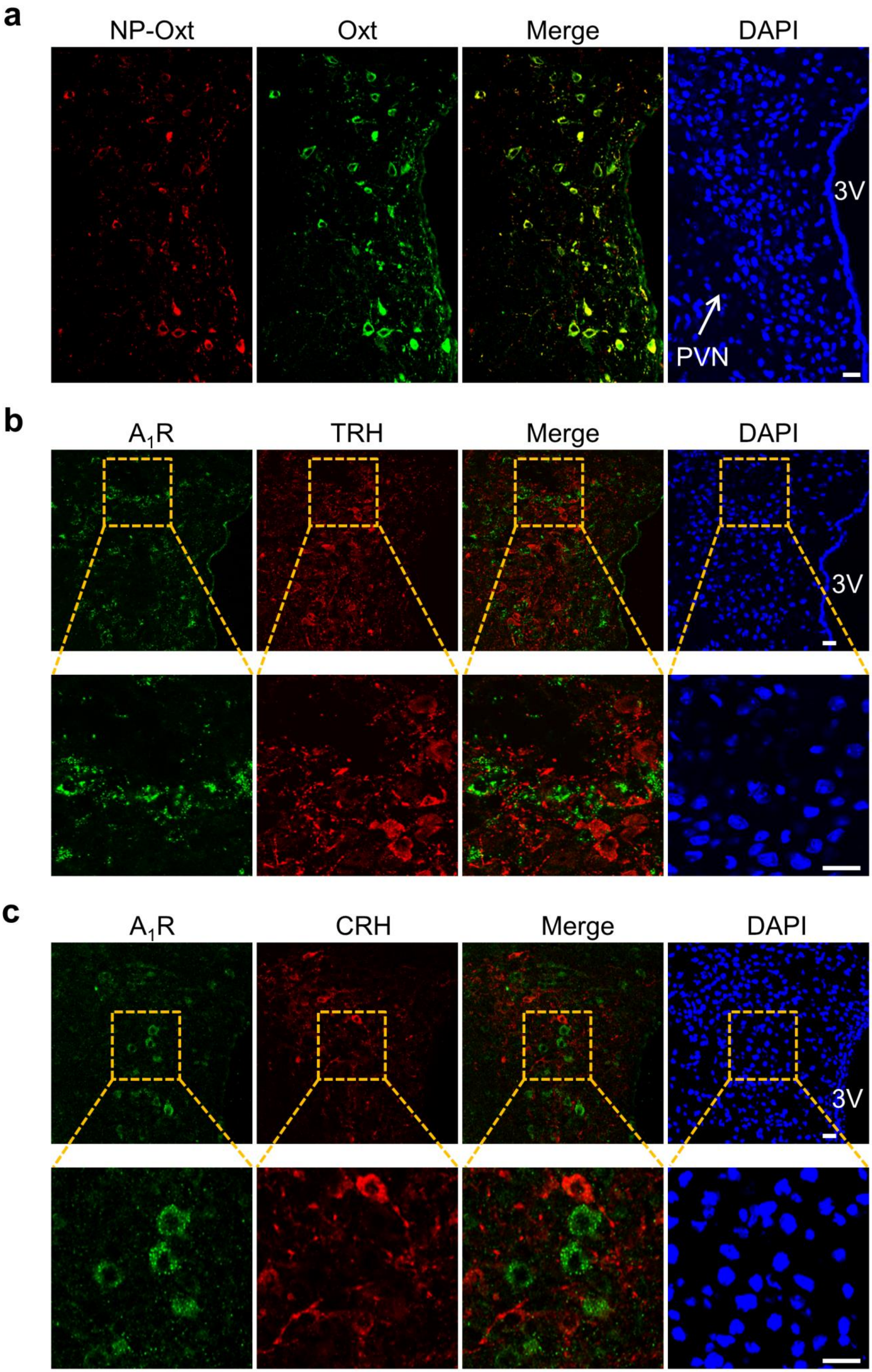
a
NP-Oxt
Oxt
Merge
DAPI
3V
PVN
b
A_1R
TRH
Merge
DAPI
3V
c
A_1R
CRH
Merge
DAPI
3V

Supplementary Fig. 11. Double immunofluorescence of NP-Oxt and Oxt, or A_1R and TRH or CRH in the mouse PVN. (**a**) Double immunofluorescence staining of NP-Oxt (red) and Oxt (green) in the PVN. (**b**-**c**) Double immunofluorescence staining of A_1R (green) and TRH (**b**) or CRH (**c**) (red) in the mouse PVN. For A_1R and TRH or CRH co-immunostaining, mice were pre-treated with colchicine (40 µg/mouse) by the i.c.v. route 2 days prior to perfusion. Cell nuclei were counterstained with DAPI (blue). **3V**, third ventricle. Scale bar, 20 µm.

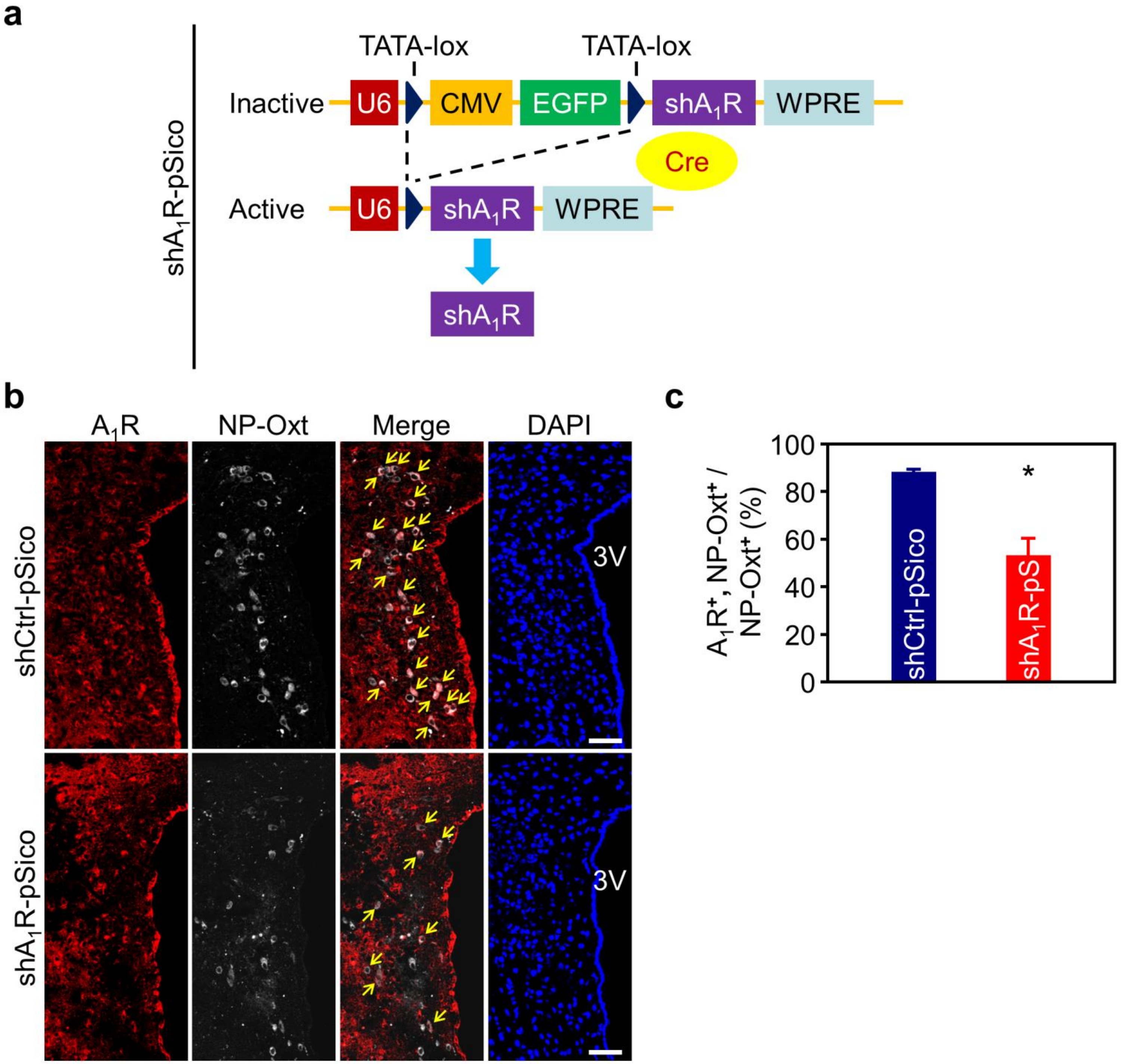

Supplementary Fig. 12. Lentivirus-mediated, Oxt neuron-specific knockdown of A_1R in mouse PVN. (**a**) Schematic diagram showing Cre-mediated, inducible expression of shRNA targeting A_1R (**shA_1R**). **U6**, human *U6* promoter. **CMV**, Cytomegalovirus promoter. **WPRE**, Woodchuck hepatitis virus post-transcriptional regulatory element. (**b**) Oxt-Cre mice were injected the control (**shCtrl-pSico**) or A_1R shRNA-expressing (**shA_1R-pSico** and **shA_1R-pS**) lentiviruses. Mice were then perfused and brains were sectioned. Double immunofluorescence staining of A_1R (red) and NP-Oxt (Alexa Fluor 633, pseudocolored white) was performed**.** Cell nuclei were counterstained with DAPI (blue). Arrows indicate NP-Oxt^+ neurons that express A_1R. **3V**, third ventricle. Scale bar, 50 μm. (**c**) Knockdown efficiency was determined by analyzing the percentage of A_1R^+, NP-Oxt^+ neurons in NP-Oxt^+ neurons in the PVN. n=3. **p*<0.05, two-tailed Student's t-test.

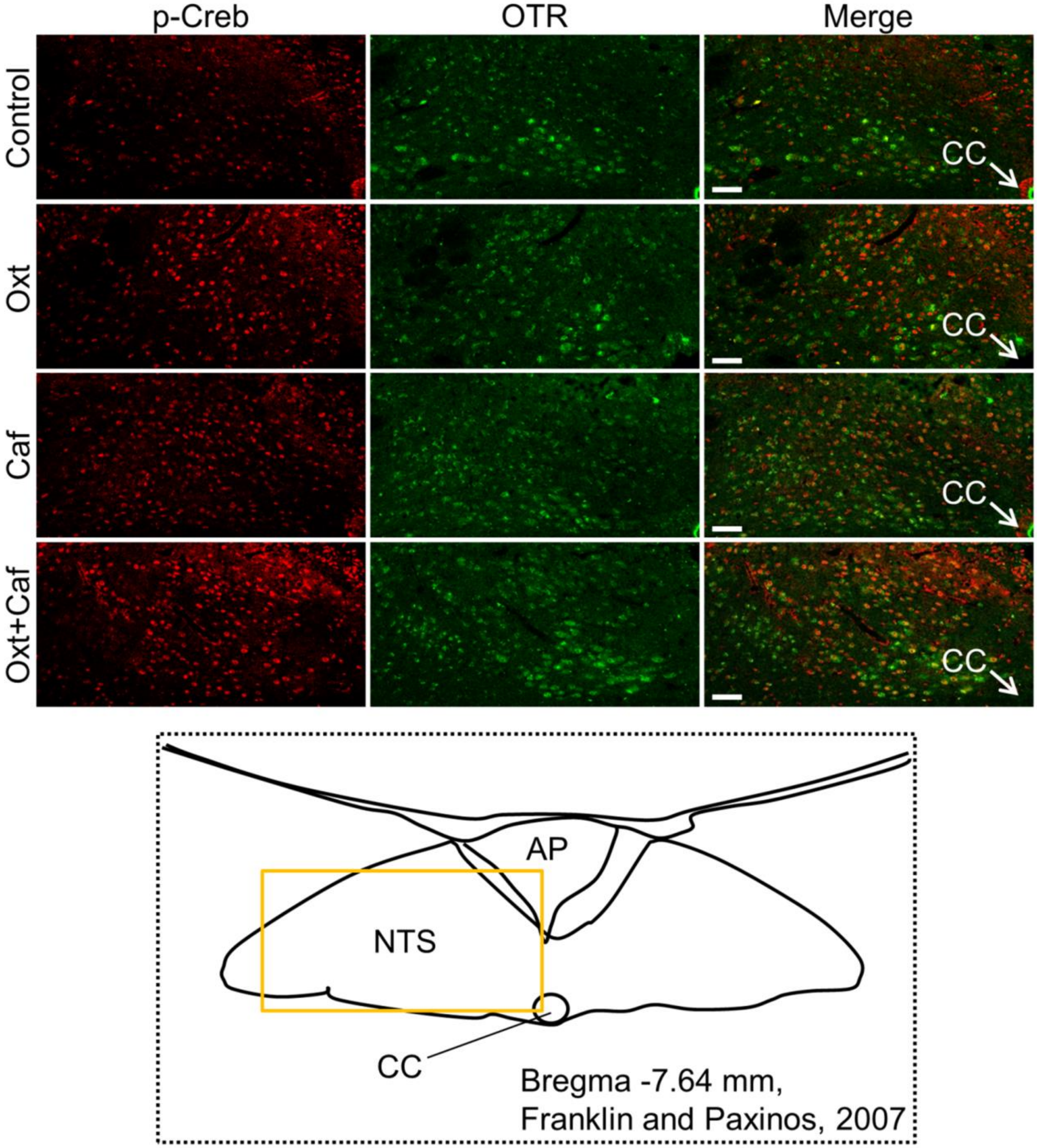

Supplementary Fig. 13. Effect of caffeine on Oxt-OTR signaling in the mouse brain. C57 BL/6 mice were i.c.v administered control aCSF or caffeine (10 µg per mouse). An hour later, mice were injected control or Oxt (1 µg per mouse) via the same route. Two hours later, mice were perfused with 4% PFA and double immunofluorescent staining of p-Creb (red) and OTR (green) in the mouse nucleus of solitary tract (**NTS**) was performed**.** Cell nuclei were counterstained with DAPI (blue). Position of the area shown in the fluorescence images in NTS was demonstrated in the schematic diagram at the bottom. **Caf**, caffeine. **OTR**, oxytocin receptor. **AP**, area postrema. **CC**, central canal. Scale bar, 50 µm.

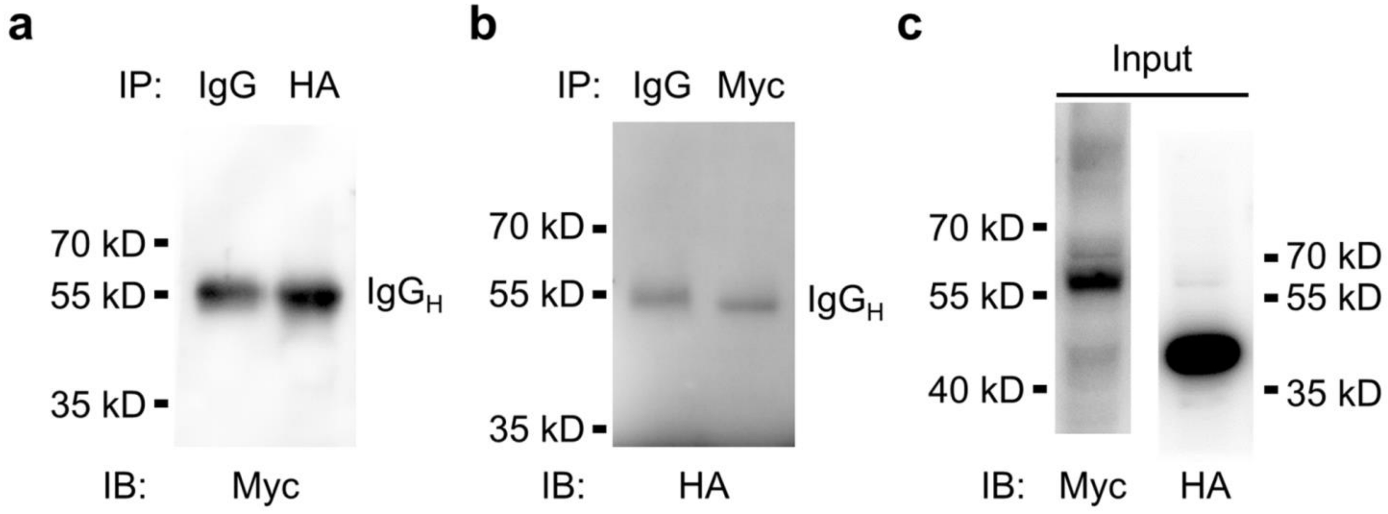

Supplementary Fig. 14. No association observed between A_1R and Oxt receptor (OTR). (**a**-**b**) Co-immunoprecipitation of HA-A_1R with Myc-OTR and vice versa. HEK293T cells were transfected with pcDNA3 HA-A_1R and Myc-OTR expressing plasmids. Cell lysates were prepared and incubated with 2 μg of non-immune IgG (as control), anti-HA (**a**) or anti-Myc (**b**) antibody. Immunoprecipitated samples were subjected to Western blot with either anti-Myc (**a**) or anti-HA (**b**) antibody as demonstrated. **IgG_H**, IgG heavy chain. **IB**, immunoblotting. **IP**, immunoprecipitation. (**c**) Protein levels of HA-A_1R and Myc-OTR in the input were examined by Western blot.

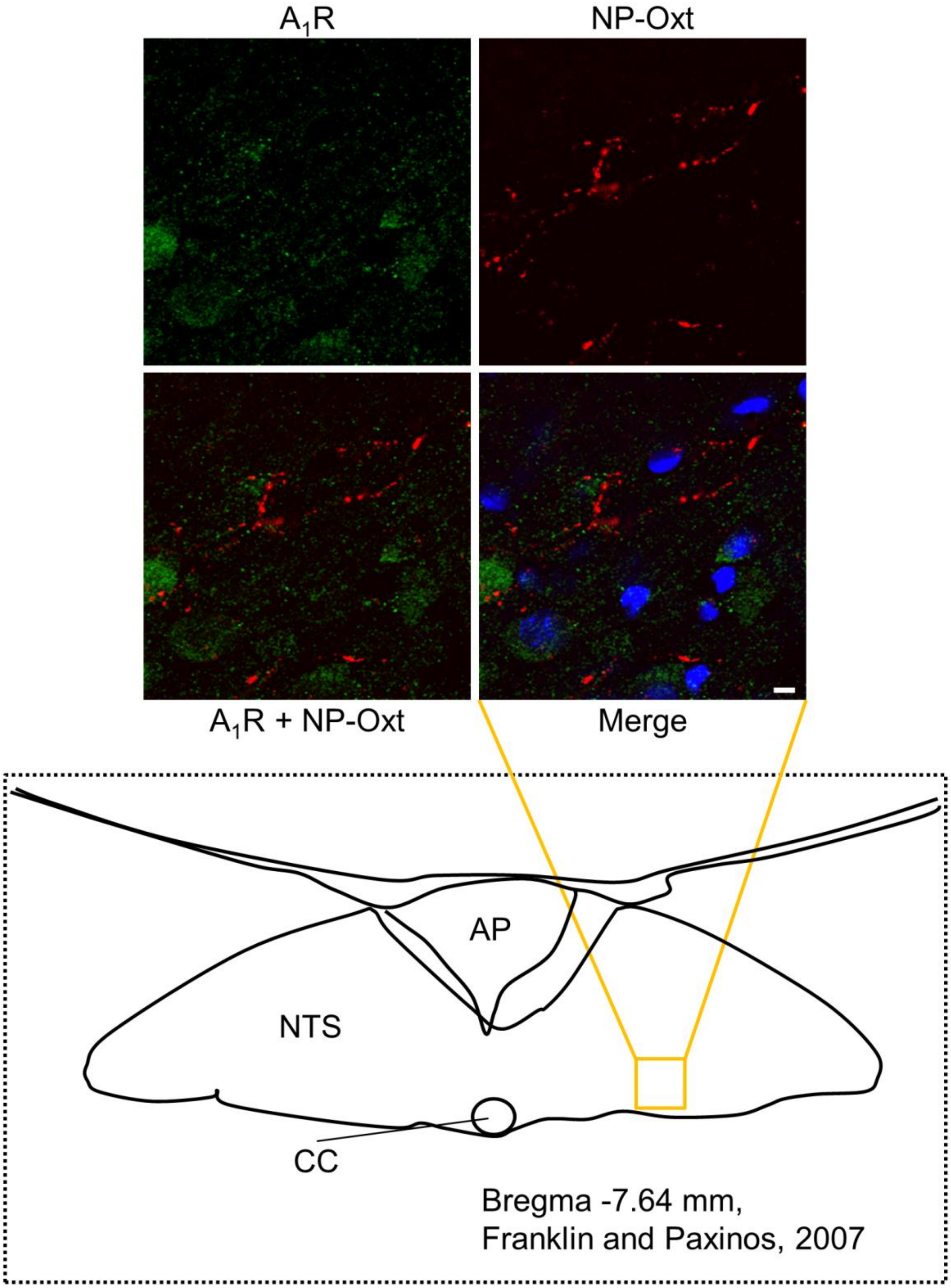

Supplementary Fig. 15. A_1R is not co-localized with NP-Oxt in the nucleus of solitary tract (NTS) of caudal brainstem. Double immunofluorescence of A_1R (green) and NP-Oxt (red) in the mouse NTS was performed. Cell nuclei were counterstained with DAPI (blue). Position of the region shown in the fluorescence images in NTS was demonstrated in the schematic diagram at the bottom. **AP**, area postrema. **CC**, central canal. Scale bar, 5 μm.

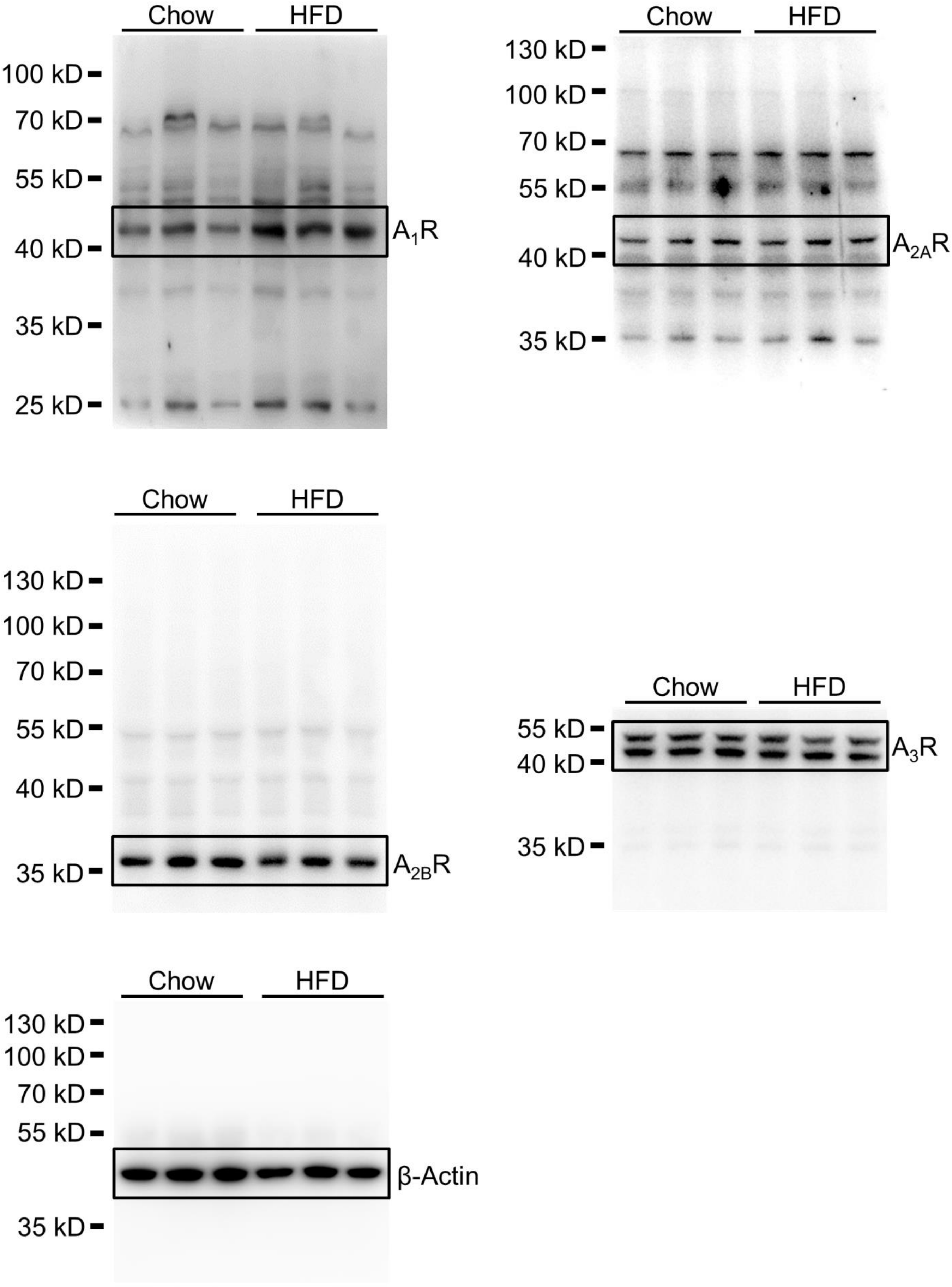

Supplementary Fig. 16. Uncropped images of the Western blots presented in Figure 1.

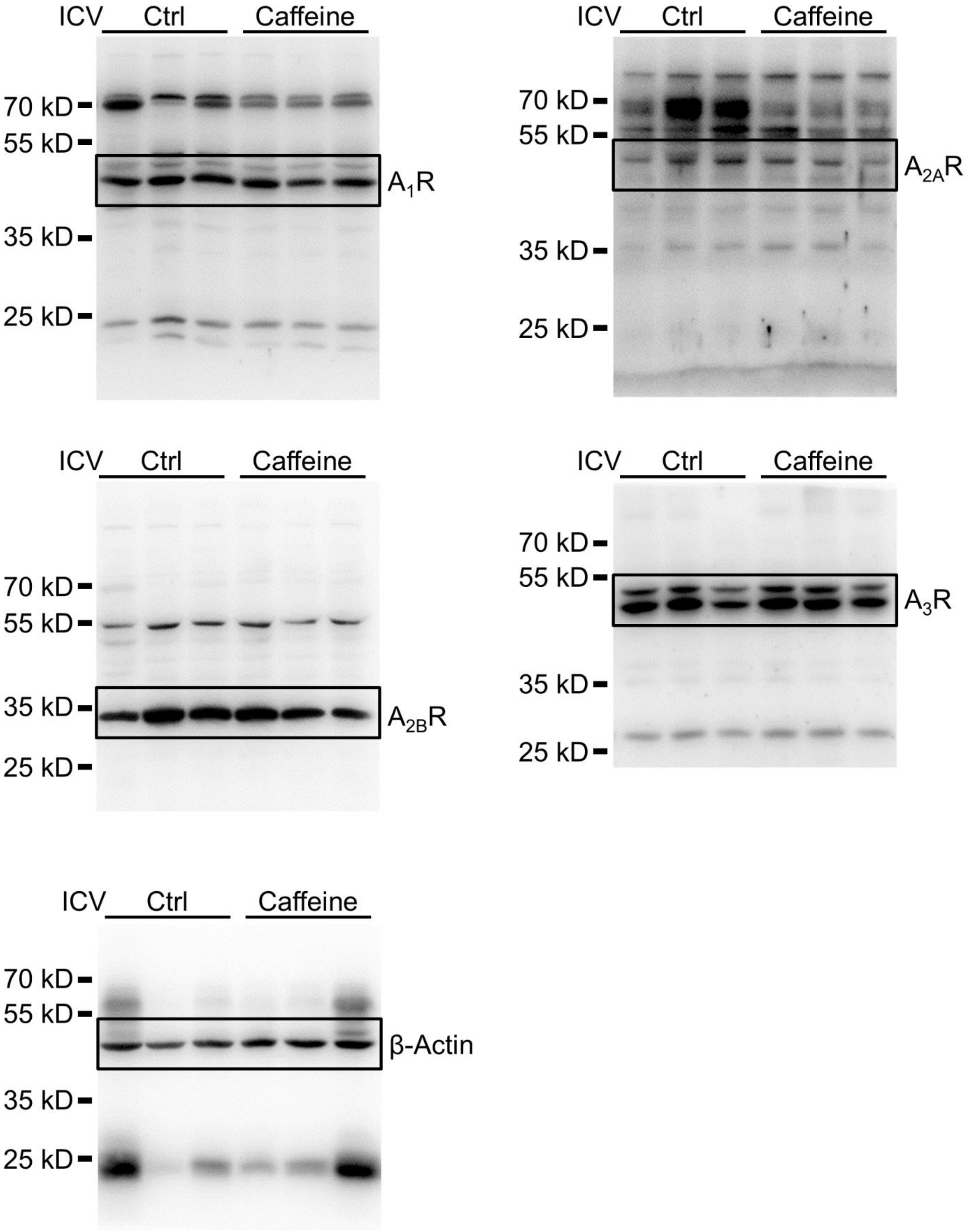

Supplementary Fig. 17. Uncropped images of the Western blots presented in Supplementary Fig. 9.

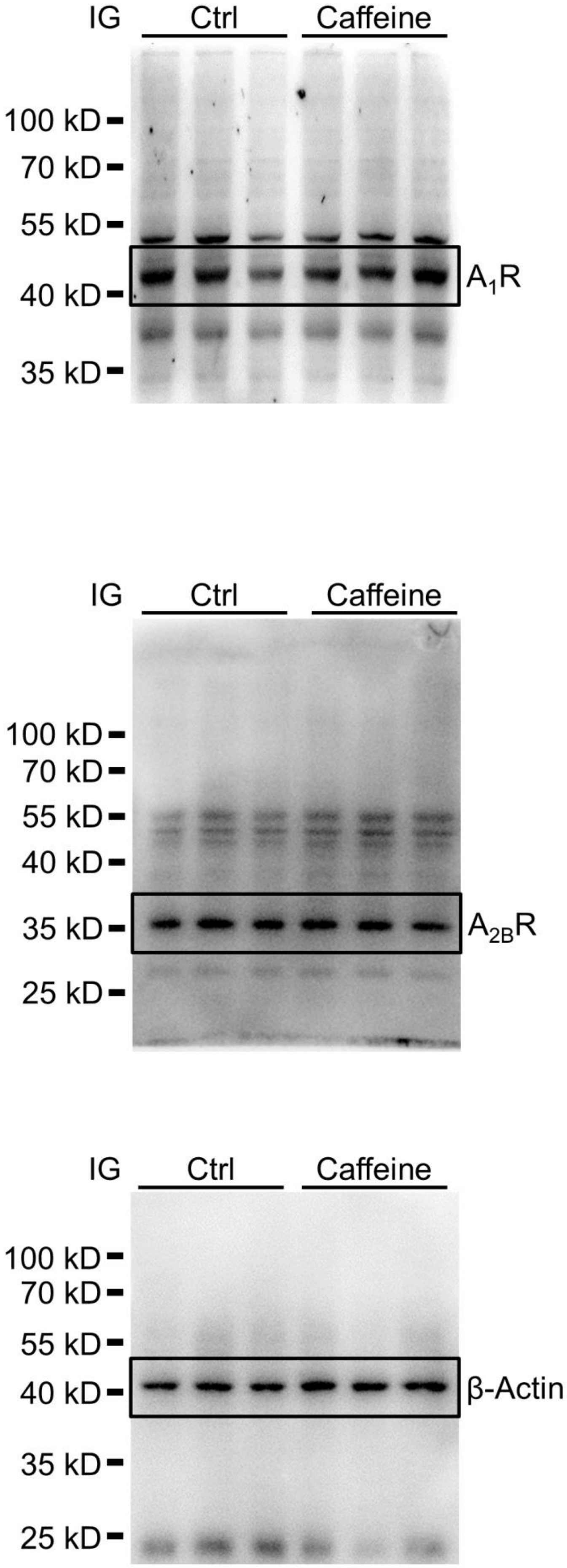

Supplementary Fig. 18. Uncropped images of the Western blots presented in Supplementary Fig. 10.

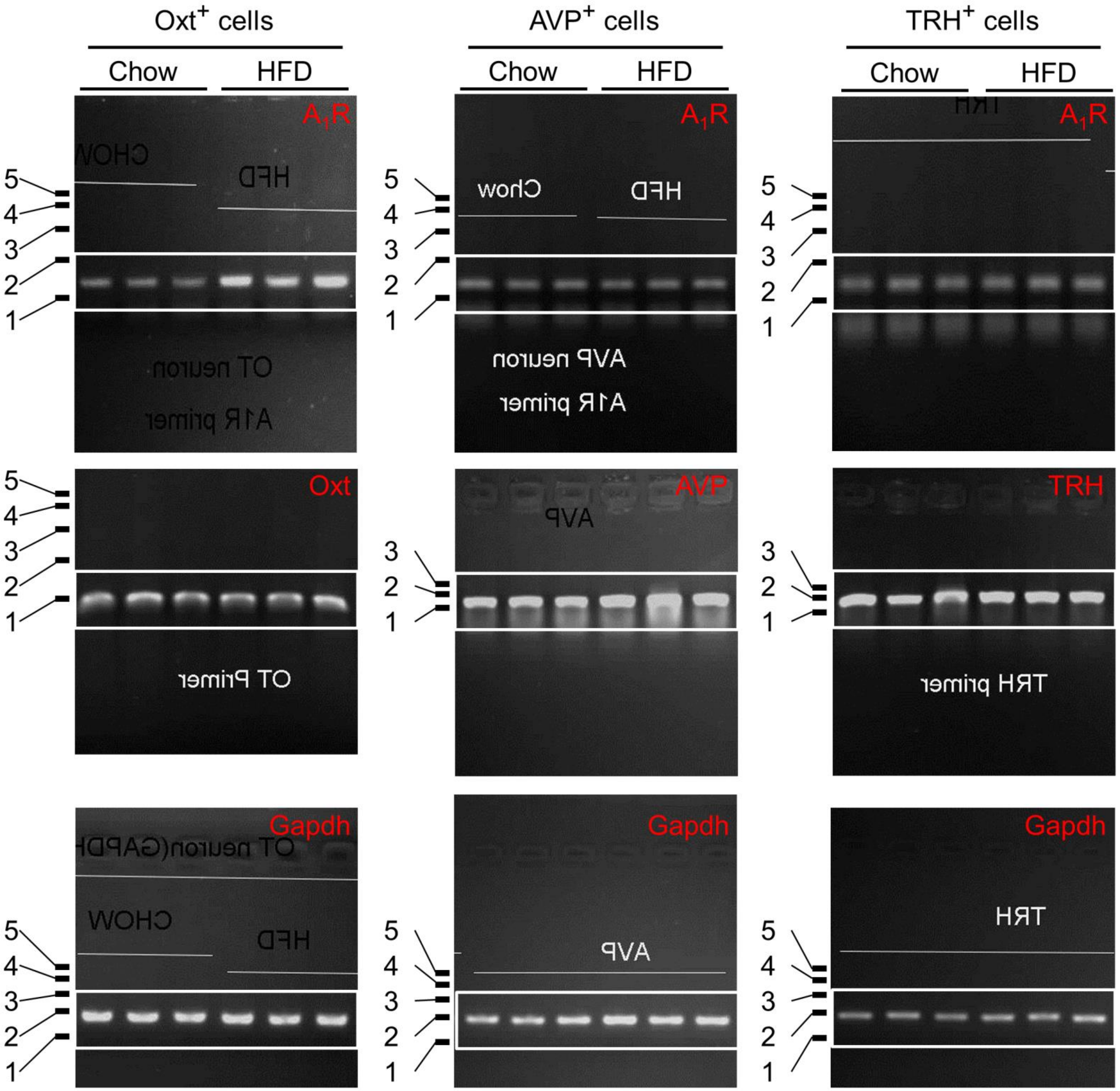

Supplementary Fig. 19. Uncropped images of the DNA agarose gels presented in Figure 6. The names of genes are shown in red font. Band size: 1, 100 bp; 2, 200 bp; 3, 300 bp; 4, 400 bp; 5, 500 bp.

Supplementary Table 1. Primary antibodies used in this study.

Name	Dilution	Species	Catalog number	Vendor
**A_1R**	IF, 1:100; WB,1:1000	Rabbit	AAR-006	Alomone labs
**A_1R**	IF, 1:50	Goat	sc-7500	Santa Cruz
**$A_{2A}R$**	IF, 1:200; WB, 1:1000	Rabbit	sc-13937	Santa Cruz
**$A_{2B}R$**	IF, 1:100; WB, 1:1000	Rabbit	bs-5900R	Bioss
**A_3R**	IF, 1:100; WB, 1:500	Rabbit	sc-13938	Santa Cruz
**c-Fos**	IF, 1:200	Rabbit	sc-7202	Santa Cruz
**c-Fos**	IF, 1:100	Goat	sc-52-G	Santa Cruz
**Hu C/D**	IF, 1:100	Mouse	A-21271	Thermo Fisher
**Neurophysin-I (NP-Oxt)**	IF, 1:50	Goat	sc-7810	Santa Cruz
**Neurophysin-II (NP-AVP)**	IF, 1:50	Goat	sc-27093	Santa Cruz
**Oxytocin**	IF, 1:400	Rabbit	20068	Immunostar
**TRH**	IF, 1:200	Rabbit	sc-366754	Santa Cruz
**CRH**	IF, 1:50	Goat	sc-1759	Santa Cruz
**AVP**	IF, 1:100	Rabbit	bs-1930R	Bioss
**OTR**	IF, 1:50	Goat	sc-8102	Santa Cruz
**HA**	IF, 1:100; WB, 1:2000	Mouse	sc-7392	Santa Cruz
**Myc**	WB, 1:1000	Mouse	66004-1-Ig	Proteintech
**p-Creb1 (Ser 133)**	IF, 1:100	Rabbit	sc-101663	Santa Cruz
**β-Actin**	WB, 1:2000	Mouse	sc-47778	Santa Cruz

Supplementary Table 2. Primer sequences.

Gene	Primer	Sequence	Purpose
**A_1R**	Forward	CTAGCTAGCATGCCGCCGTACATCT	Cloning. To generate A_1R-Lenti.
	Reverse	CTAGCTAGCGTAGGGCCGGGATTCTCCTCCACGTCACCGCATGTTAGAAGACTTCCTCTGCCCTCGTCATCAGCTTTCTCCTCTG	
**A_1R**	Forward	TGTCGCTGGCCCTCATCCTCTTCAAGAGAGAGGATGAGGGCCAGCGACTTTTTTC	Cloning. To generate shA_1R-Lenti and shA_1R-pSico.
	Reverse	TCGAGAAAAAAGTCGCTGGCCCTCATCCTCTCTCTTGAAGAGGATGAGGGCCAGCGACA	
**A_1R**	Forward	CCCAAGCTTATGTACCCATACGATGTTCCAGATTACGCTATGCCGCCGTACATCTCG	Cloning. To generate pcDNA3 HA-A_1R.
	Reverse	CGGAATTCCTAGTCATCAGCTTTCTCCTC	
**A_1R**	Forward	GCAAGAGGCGGACATCACA	qRT-PCR
	Reverse	TCACTCACCCTAGAAGCCATACTC	
**$A_{2A}R$**	Forward	CTTTGTCCTGGTCCTCACGC	qRT-PCR
	Reverse	TCGCAATGATGCCCTTCG	
**$A_{2B}R$**	Forward	TCTTCCTCGCCTGCTTCGT	qRT-PCR
	Reverse	CTCGTGTCCCAGTGACCAAAC	
**A_3R**	Forward	TGTGCCACTTCCGTTCCG	qRT-PCR
	Reverse	CCAGACAGGTTTTGACTGAGCTTA	
**Gapdh**	Forward	CCCACTCTTCCACCTTCGAT	qRT-PCR
	Reverse	CCTCTCTTGCTCAGTGTCCT	
**Oxt**	Forward	TTGGCTTACTGGCTCTGACC	qRT-PCR
	Reverse	GGGAGACACTTGCGCATATC	
**AVP**	Forward	GCTGCCAGGAGGAGAACTAC	qRT-PCR
	Reverse	AAAAACCGTCGTGGCACTCG	
**TRH**	Forward	TCCTGGATCACAAAACGCCA	qRT-PCR
	Reverse	CTTGTCTTGGTTGGCACGTC	
**Ucp1**	Forward	CATGGGATCAAACCCCGCTA	qRT-PCR
	Reverse	TTAGGGGTCGTCCCTTTCCA	
**Pgc1b**	Forward	CTCTGACACGCAGGGTGG	qRT-PCR
	Reverse	AGTCAAAGTCACTGGCGTCC	
